# PP2Acα Deficiency in Vascular Smooth Muscle Cells Accelerates Aortic Aneurysm and Dissection by Regulating KLF4 Phosphorylation and Ubiquitination

**DOI:** 10.1002/advs.202500102

**Published:** 2025-07-28

**Authors:** Wei‐Peng Hu, Ze‐Yu Cai, Qing‐Le Li, Tao Zhang, Xiang‐Yu Chu, Chang Wang, Qing‐Yi Zhang, Rong Qi

**Affiliations:** ^1^ Department of Pharmacology School of Basic Medical Sciences Peking University Health Science Center 38 Xueyuan Road, Haidian District Beijing 100191 China; ^2^ State Key Laboratory of Vascular Homeostasis and Remodeling Peking University Beijing 100191 China; ^3^ State Key Laboratory of Natural and Biomimetic Drugs Peking University Beijing 100191 China; ^4^ NHC Key Laboratory of Cardiovascular Molecular Biology and Regulatory Peptides Peking University Beijing 100191 China; ^5^ Beijing Key Laboratory of Molecular Pharmaceutics and New Drug Delivery Systems Peking University Beijing 100191 China; ^6^ Department of Physiology and Pathophysiology School of Basic Medical Sciences Peking University Health Science Center Beijing 100191 China; ^7^ Department of Vascular Surgery Peking University People's Hospital Beijing 100044 China

**Keywords:** aortic aneurysm/dissection, kruppel‐like factor 4, phenotypic switching, protein phosphatase 2A, vascular smooth muscle cell

## Abstract

Aortic aneurysm and dissection (AAD) are life‐threatening cardiovascular diseases with limited effective medical treatments. Protein phosphatase 2A (PP2A), the most abundant serine/threonine phosphatase in eukaryotes, is pivotal in regulating intracellular signaling. This study investigates the role of PP2A in the pathogenesis of AAD. Analysis of available datasets revealed that PP2Acα is the most significantly downregulated PP2A subunit in human and mouse aneurysm tissues. Vascular smooth muscle cell (VSMC)‐specific PP2Acα knockout exacerbates β‐Aminopropionitrile (BAPN)‐induced aortic dissection and elastase‐induced abdominal aortic aneurysm in mice. Collagen‐based contraction assays, Western blot, and gelatin zymography confirmed that the deficiency of PP2Acα results in decreased contractility, contractile markers, and elevated production of matrix metallopeptidase 2 (MMP2) in VSMCs. Furthermore, PP2Acα deficiency promoted VSMC phenotypic switching through stabilizing Kruppel‐like factor 4 (KLF4). Mechanistically, PP2Acα binds to and dephosphorylates protein kinase B 1 (AKT1), thereby reducing phosphorylation of the AKT1 substrate KLF4 at Thr398. The deficiency of PP2Acα diminishes KLF4 phosphorylation‐dependent ubiquitination and degradation, leading to the suppression of VSMC contractile gene transcription. The findings underscore a critical role for PP2Acα in regulating VSMC phenotypic switching and AAD progression by controlling KLF4 phosphorylation and ubiquitination, offering novel insights into the molecular pathogenesis underlying AAD.

## Graphical abstract

Vascular smooth muscle cells (VSMCs)‐specific deficiency of PP2Acα exacerbates aortic aneurysm and dissection (AAD) by promoting phenotypic switching through AKT1‐mediated phosphorylation and stabilization of Kruppel‐like factor 4 (KLF4). Reduced PP2Acα enhances KLF4 ubiquitination resistance, suppressing VSMC contractile genes and accelerating aortic degeneration, unveiling a novel PP2A‐AKT1‐KLF4 axis as a critical determinant in AAD pathobiology.

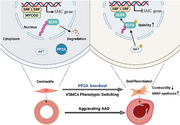

## 1 Introduction

Aortic aneurysm and dissection (AAD) is an aggressive condition characterized by either a localized or diffuse dilation of the aorta exceeding 30 mm or a tear in the intimal layer leading to false lumen formation^[^ ^1^ ^]^ Without prompt diagnosis and intervention, AAD typically progresses, with a mortality rate exceeding 80% upon aortic rupture. Several risk factors, such as sex, age, smoking, hypertension, and hypercholesterolemia, have been identified as contributors to AAD development.^[^ ^1^ ^]^ Although endovascular repair and open surgery remain the only current treatment options for aneurysms, the prognosis remains poor, and mortality rates are still high.^[^ ^2^ ^]^ There is an urgent need to understand the molecular mechanisms driving AAD pathogenesis to develop effective prevention strategies.

In healthy vessel walls, Vascular smooth muscle cells (VSMCs) exhibit a contractile phenotype that expresses unique contractile proteins required for vascular tone regulation to maintain arterial diameter.^[^ ^3^, ^4^ ^]^ VSMCs are highly malleable and can undergo reversible contractile‐synthetic phenotype switching in response to pathologic activation.^[^ ^5^ ^]^ This process is characterized by decreased contractile protein expression and increased expression of matrix metallopeptidases (MMPs) and pro‐inflammatory factors, which degrade the extracellular matrix and facilitate inflammatory cell infiltration, contributing to dilatation and degeneration of the aortic wall. Therefore, a better understanding of the mechanisms underlying VSMC phenotype switch during AAD progression could reveal novel therapeutic strategies for AAD treatment. Protein phosphorylation, a common post‐translational modification, is very important in the regulation of VSMC phenotype. For example, MAP kinase kinase‐2/3 phosphorylation at S153/61 by bestrophin3 deficiency impedes VSMC phenotypic regulation.^[^ ^6^ ^]^ Forkhead Box O4 (FOXO4) is phosphorylated and inactivated by protein kinase B (AKT) signaling, which increases FOXO4 cytoplasmic localization and the expression of differentiation genes.^[^ ^7^ ^]^ Protein phosphorylation is modulated by the action of protein kinases and protein phosphatases. For many years, kinases have been regarded as the primary regulators of coordinated protein phosphorylation and viewed as drug targets. The activations and functions of many kinases, as well as their substrates, in the regulation of VSMC phenotype have been well understood.^[^ ^8^, ^9^ ^]^ Although the dephosphorylating function of phosphatases suggests that they have the same importance as kinases, phosphatases have been generally ignored until recently.^[^ ^10^ ^]^ From a therapeutic standpoint, kinase inhibition is limited to the de novo phosphorylation of unphosphorylated amino acids, whereas drug‐driven increases in phosphatase activity are anticipated to dephosphorylate existing phosphorylation sites. However, studies investigating the effect of phosphatase in VSMC phenotypic regulation are currently lacking.

Protein phosphatase 2A (PP2A) is the most widely distributed and abundant phosphatase expressed in eukaryotes, which accounts for ≈55–70% of all serine/threonine phosphatase phosphosites and is essential for regulating a multitude of cellular processes, including cell cycle, apoptosis and growth.^[^ ^11^ ^]^ PP2A exists mainly as a trimeric holoenzyme consisting of three types of subunits: catalytic subunit C, scaffolding subunit A and regulatory subunit B.^[^ ^12^ ^]^ The C subunits are the core component responsible for PP2A holoenzyme activity and have two isoforms (PP2Acα and PP2Acβ). The more active promoter region of PP2Acα results in the expression of PP2Acα being 10‐fold higher than PP2Acβ in most cell types.^[^ ^13^ ^]^ PP2A has been shown to be involved in a variety of physiological and pathophysiological processes, including tumorigenesis, pulmonary fibrosis, cardiac hypertrophy and angiogenesis.^[^ ^14^, ^15^, ^16^, ^17^ ^]^ Previous studies reported that PP2A activation inhibits VSMC migration and proliferation,^[^ ^18^, ^19^ ^]^ suggesting a potential link between PP2A and VSMC phenotypic switch. However, the role and molecular mechanisms for PP2A in regulating VSMC phenotypic switch and AAD remain largely to be elucidated.

In this study, by using PP2Acα VSMC‐specific deletion mice, we observed that PP2Acα deficiency accelerates both BAPN‐induced AAD and elastase‐induced abdominal aortic aneurysm progression in mice. Mechanistically, PP2A deficiency inhibits phosphorylation‐dependent ubiquitination and degradation of KLF4 by activating AKT, thus promoting the phenotype switch in VSMCs from contractile to secretory. Collectively, our study revealed a critical role for PP2Acα in maintaining VSMC contractile phenotypes and limiting AAD formation.

## 2 Results

### 2.1 PP2Acα is Downregulated in Murine AAD Models

**Figure 1:**
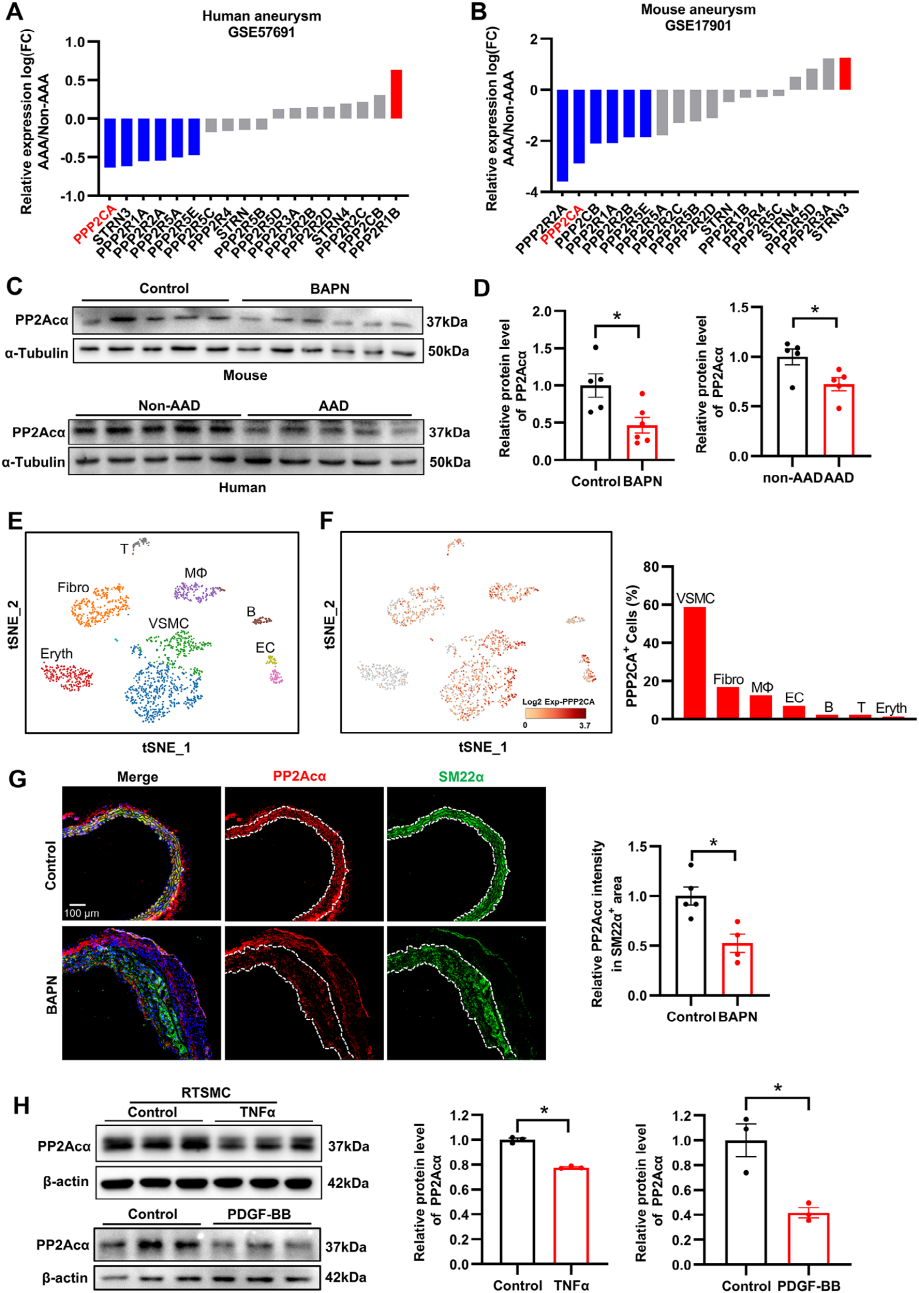
PP2Acα is downregulated in human and murine aneurysm tissues. A) Relative mRNA expression of 18 PP2A subunits in human (GSE57691) and B) murine aneurysm tissues (GSE17901). C) Representative Western blot images and D) quantification analysis of PP2Acα protein in mouse aortas after BAPN (0.5% in drinking water) treatment for 2 weeks and in aortic tissues from non‐AAD and AAD patients. *N* = 5‐6, Student's *t* test. E) t‐distributed stochastic neighbor embedding (tSEN) plots of single cells from mouse abdominal aortas (GSE152583). VSMC, vascular smooth muscle cells; EC, endothelial cells; MΦ, macrophages; Fibro, fibroblasts; B, B cells; T, T cells; DC, dendritic cells; Eryth, erythrocytes. F) Visualization and quantification of PPP2CA expression amongst seven lineages. G) Representative images of triple immunofluorescence for PP2Acα, SM22α, and DAPI (4′,6‐diamidino‐2‐phenylindole), and quantification of PP2Acα expression in the SM22α^+^ area of mouse aortas after BAPN treatment for 4 weeks. *N* = 4‐5, Student's *t* test. H) Representative Western blot images and quantification analysis of PP2Acα protein in primary rat aortic smooth muscle cells (RTSMC) exposed to TNFα (20 µg/mL) or PDGF‐BB (20 ng/mL) for 48 h. *N* = 3, Student's *t* test. ^*^ *P* < 0.05.

To investigate whether the expression of subunits is modulated in the aortic tissues of AAD patients, we conducted a re‐analysis of the RNA‐sequencing dataset GSE57691. The analysis revealed that most of the significantly altered PP2A subunits were downregulated in aneurysmal tissues. Among these, PP2Acα, which is primarily responsible for the catalytic activity of the PP2A holoenzyme, showed the most pronounced downregulation among all subunits (**Figure** **1**A). Similar results were observed in the mouse aneurysmal tissues (GSE17901) (Figure 1B). The decreased PP2Acα expression was validated in aneurysmal tissues from patients with AAD and rodent models with β‐Aminopropionitrile (BAPN)‐induced mouse AAD (Figure 1C,D). Immunofluorescence staining further confirmed the reduced expression of PP2Acα in human AAD tissues (Figure S1A, Supporting Information). Consistent with the decreased expression of PP2Acα, the activity of PP2A is reduced in mouse AAD tissues (Figure S1B, Supporting Information). To observe the abundance of PP2Acα within vascular wall cell lineages, we re‐analyzed the single‐cell RNA sequencing (scRNA‐seq) dataset of mouse abdominal aorta (GSM4618435). Screening of seven major lineages revealed that PP2Acα was predominantly expressed in VSMCs (Figure 1E,F). Additionally, immunofluorescence staining of mouse aortic tissues showed a significant reduction of PP2Acα in smooth muscle protein 22‐α (SM22α)‐positive VSMCs within AAD tissues compared to normal aortic tissues (Figure 1G). Immunohistochemical analysis further confirmed that the expression of PP2Acα was lower in the vascular media of AAD tissues compared to the intima and adventitia (Figure S1C, Supporting Information). Thus, reduced PP2Acα expression was also verified in VSMCs treated with tumor necrosis factor α (TNFα) or platelet‐derived growth factor‐BB (PDGF‐BB) (Figure 1H). Collectively, these findings indicate that PP2Acα is significantly downregulated in AAD tissues, with its reduced expression appearing to be primarily localized to VSMCs.

### 2.2 Deficiency of PP2Acα in VSMC Aggravated AAD Formation

**Figure 2:**
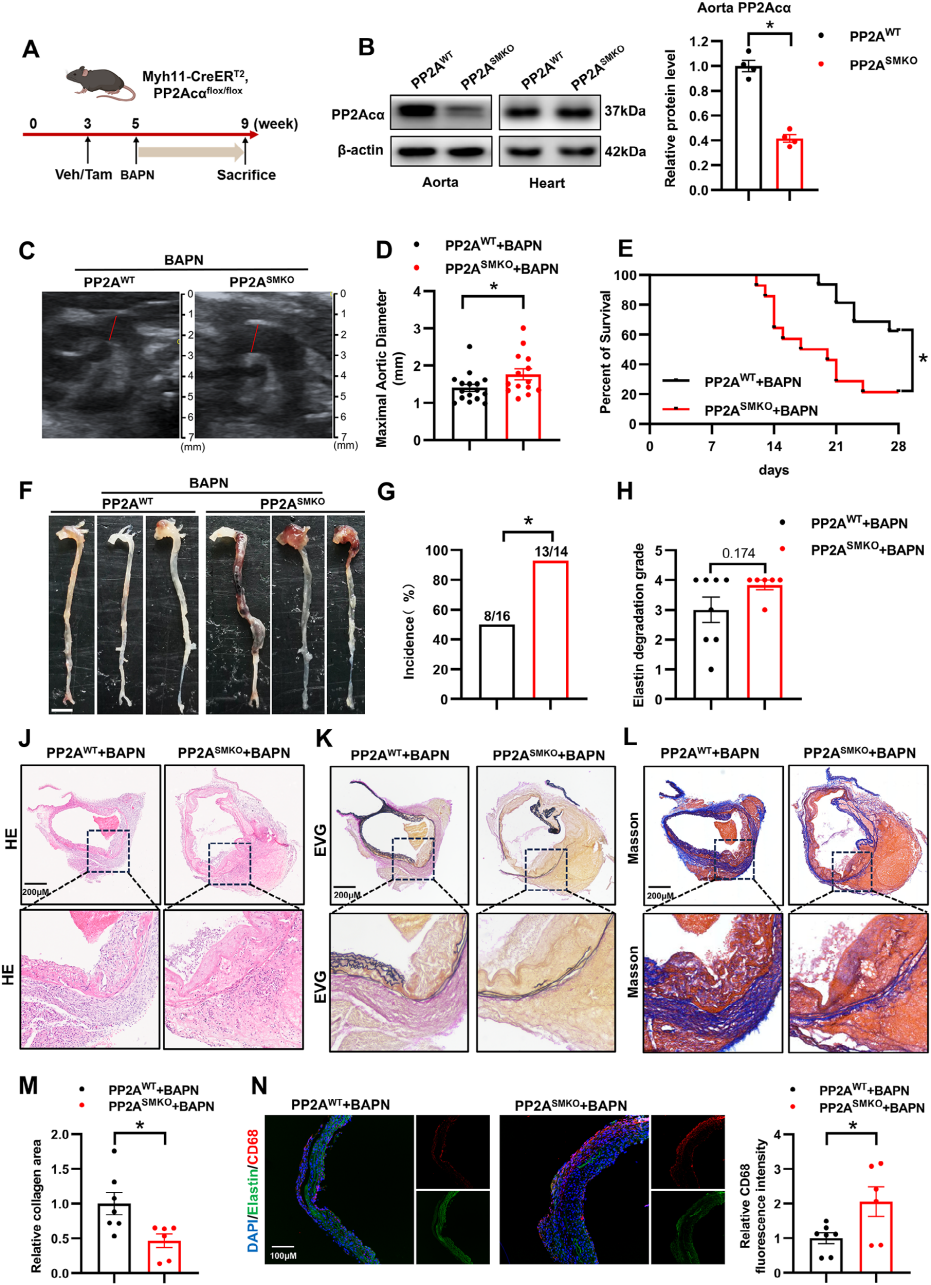
PP2Acα deficiency in VSMCs aggravates BAPN‐induced AAD development. A) Experimental design. Three‐week‐old *Myh11‐CreER^T2^, PP2Acα^flox/flox^* mice were intraperitoneally injected daily with oil or tamoxifen (75 mg kg^−1^) for 5 consecutive days. After a rest for 9 days, the five‐week‐old mice were fed with BAPN (0.5% in drinking water) for 4 weeks to establish the AAD model. B) Representative Western blot images and quantitative analysis of PP2Acα expression in aortas from the 12‐week‐old PP2A^WT^ and PP2A^SMKO^ mice. *N* = 4, Student's *t* test. C) Representative aorta ultrasound images of PP2A^WT^ and PP2A^SMKO^ at 28 days after BAPN treatment. D) Maximal aortic diameters, E) The survival curves, F) Representative pictures of whole aortas, and G) Aneurysm incidence of PP2A^WT^ and PP2A^SMKO^ mice after BAPN treatment for 28 days. Scale bars = 2.5 mm. *N* = 14‐16, Student's *t* test for panel D, Kaplan–Meier method, and compared by log‐rank tests for panel E, Chi‐square test for panel G. H) Elastin fragmented score in the thoracic aortas from PP2A^WT^ and PP2A^SMKO^ mice after BAPN treatment for 28 days. *N* = 6‐8, Mann–Whitney test. J–L) Representative images of histological staining with hematoxylin and eosin (HE), Verhoeff's Van Gieson (EVG), and Masson Trichrome (Masson) in aortic sections of BAPN‐treated PP2A^WT^ and PP2A^SMKO^ mice. Scale bars = 200 µm. M) Quantification of collagen deposition in the aortic sections of BAPN‐treated PP2A^WT^ and PP2A^SMKO^ mice. Student's *t* test. N) Representative images of CD68 immunofluorescence and quantification of CD68 intensity in the aortas from PP2A^WT^ and PP2A^SMKO^ mice after BAPN treatment. *N* = 6‐7, Student's *t* test. ^*^ *P* < 0.05.

Next, to explore whether PP2Acα in VSMC was involved in the development of AAD, we generated the tamoxifen‐induced VSMC‐specific PP2Acα knockout (PP2A^SMKO^) mice (Figure S2A, Supporting Information). Three‐week‐old *myosin heavy chain 11 (* *Myh11)‐CreER^T2^. PP2Acα^flox/flox^* mice were injected with tamoxifen (75 mg kg^−1^, intraperitoneal) for 5 consecutive days to induce PP2Acα deletion in VSMCs. After 14 days, PP2Acα protein level markedly decreased in the aortas, but not in the hearts of PP2A^SMKO^ mice (**Figure** **2**B). We then assessed the physiological characteristics of 12‐week‐old PP2A^WT^ and PP2A^SMKO^ mice, and no measurable differences were found in blood pressure or aortic wall structure (Figure S2B,C, Supporting Information). Next, five‐week‐old PP2A^WT^ and PP2A^SMKO^ mice were administrated with BAPN (0.5% in drinking water) treatment for 28 days to induce AAD. As a result, the maximal aortic diameters were significantly greater in PP2A^SMKO^ mice compared to PP2A^WT^ mice (Figure 2C,D). Survival analysis showed that the aneurysm ruptures were earlier in the PP2A^SMKO^ mice (begin rupturing from 12 days after aneurysm induction) than in the PP2A^WT^ mice (begin rupturing from 19 days after aneurysm induction) (Figure 2F), and the aneurysm incidence was 50% for PP2A^WT^ mice, but 92.8% for PP2A^SMKO^ mice (Figure 2F,G). Elastic van gieson staining of aortic sections from BAPN‐induced PP2A^SMKO^ mice showed more severe elastic fiber breaks compared with that in PP2A^WT^ (Figure 2H,K). Hematoxylin and eosin (HE) and Masson staining revealed that PP2A^SMKO^ mice exhibited marked thickening of the aortic wall compared to PP2A^WT^ controls (Figure 2J), along with enhanced degradation of collagen fibers (Figure 2L,M). Furthermore, VSMC PP2Acα deficiency exacerbated BAPN‐induced macrophage infiltration in the aortas, as evidenced by cluster of differentiation 68 (CD68) staining (Figure 2N). To further interrogate the role of PP2A in AAA, a well‐established elastase‐induced AAA mouse model was used. Similarly, the abdominal aortas were more significantly expanded in PP2A^SMKO^ mice than in PP2A^WT^ mice (Figure S3A,B, Supporting Information). Histological analyses showed that the aneurysmal vascular remodeling of abdominal aortas was severe in the PP2A^SMKO^ mice (Figure S3C, Supporting Information). Collectively, these results suggested that VSMC‐specific PP2A deficiency aggravated BAPN‐induced AAD and elastase‐induced AAA development in mice.

Additionally, to evaluate the therapeutic potential of PP2A in AAD, wild‐type C57BL/6J mice received daily intraperitoneal injections of DT061 (a specific PP2A agonist), beginning on day 7 after BAPN administration and continuing treatment for 14 days until the end of the experiments (Figure S4A, Supporting Information). DT061 treatment reduced the aortic dilatation in the BAPN‐treated mice (Figure S4B,C, Supporting Information) and attenuated the BAPN‐induced incidence of AAD at 21 days from 80% (8/10) to 40% (4/10), improved the survival rate from 30% (3/10) to 80% (8/10) (Figure S4D,E, Supporting Information). EVG and HE staining revealed that DT061 treatment significantly attenuated BAPN‐induced elastin fragmentation and aortic wall thickening (Figure S4F–H, Supporting Information).

### 2.3 PP2A Deficiency Leads to Downregulation of VSMC Contractile Phenotype‐Related Pathways

**Figure 3:**
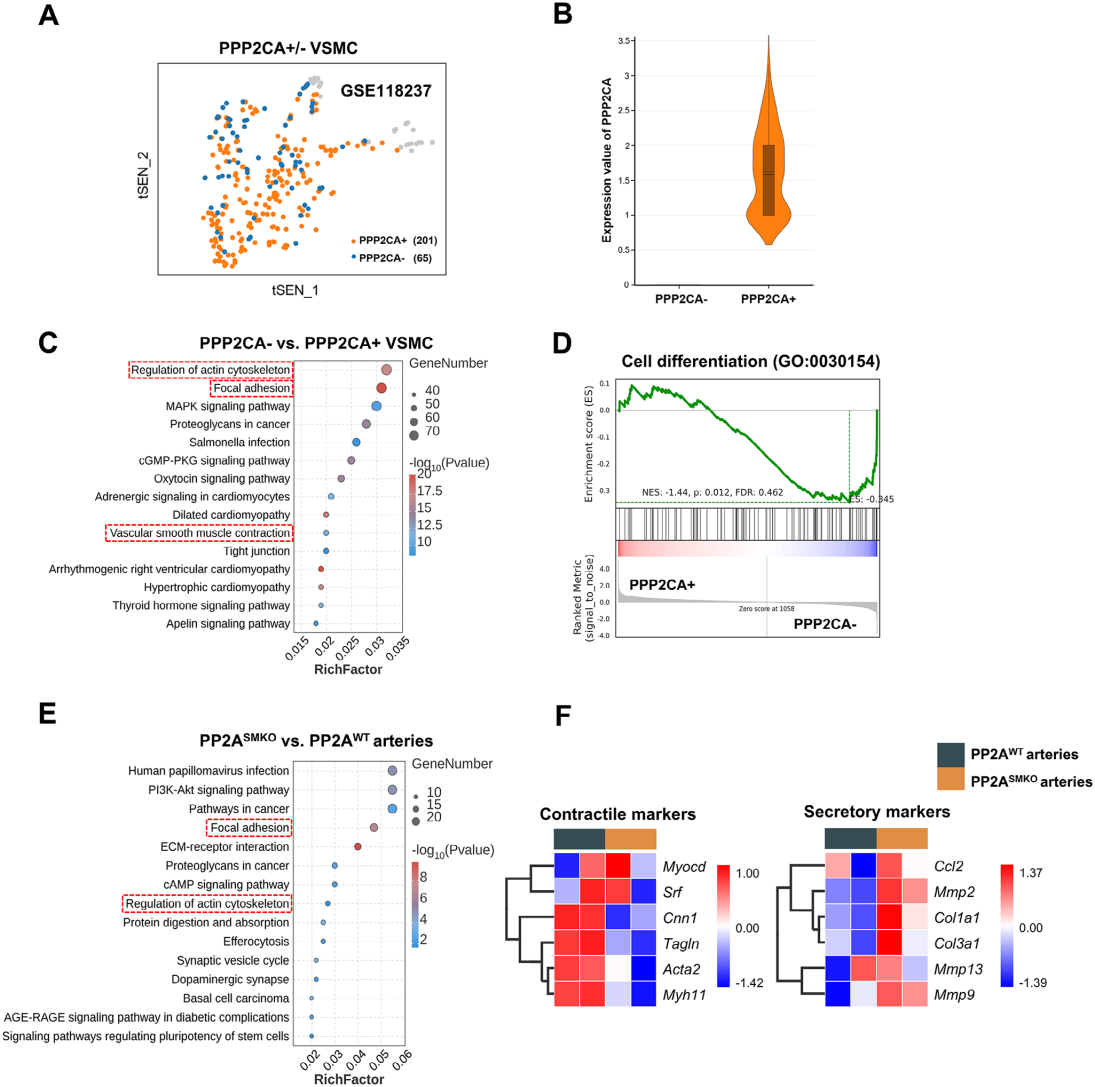
VSMC contractile phenotype‐related pathways are decreased in the PPP2CA‐ cluster in VSMCs and aortas from PP2A^SMKO^ mice. A) tSEN plots of the PPP2CA+ and PPP2CA‐ clusters in VSMCs from murine aneurysms (GSE118237). B) The expression values of PPP2CA in the PPP2CA+ and PPP2CA‐ clusters. C) KEGG pathway enrichment analysis of downregulated DEGs from the PPP2CA‐ clusters compared to the PPP2CA+ cluster. D) GSEA of DEGs from the PPP2CA+ cluster compared to the PPP2CA‐ cluster. E) KEGG pathway enrichment analysis of downregulated DEGs in aortas from PP2A^SMKO^ mice. F) Heatmap of contractile markers and secretory markers. Heatmap color indicates Z‐score.

To determine the mechanisms underlying PP2Acα deficiency‐mediated AAD pathology, we re‐analyzed a published single‐cell transcriptome dataset of mouse aneurysmal samples (GSE118237) and separated VSMCs into high and low PP2Acα subpopulations (PPP2CA+ and PPP2CA‐) (**Figure** **3**A,B). Functional enrichment analysis of Kyoto Encyclopedia of Genes and Genomes (KEGG) analysis showed that VSMC contractile phenotype‐related pathways, including focal adhesion, regulation of actin cytoskeleton and vascular smooth muscle contraction, were remarkably downregulated in the PPP2CA‐ cluster (Figure 3C). Furthermore, gene set enrichment analysis (GSEA) confirmed that genes related to cell differentiation were downregulated in the PPP2CA‐ cluster compared to the PPP2CA+ cluster (Figure 3D). To further confirm the role of PP2Acα in the VSMC phenotypic switch, we analyzed mRNA profiles in aortas from the PP2A^WT^ or PP2A^SMKO^ mice. The data revealed that 425 differentially expressed genes in PP2A^SMKO^ tissues compared with those in PP2A^WT^ tissues (|log2FC| > 0.5 and FDR < 0.05): 192 upregulated and 231 downregulated transcripts (Figure S5, Supporting Information). KEGG verified that the top differentially downregulated genes were enriched in the VSMC contractile phenotype‐related pathways, including focal adhesion and regulation of actin cytoskeleton (Figure 3E). The heatmap showed that VSMC contractile markers such as acta2, cnn1, tagln and myh11 were decreased in aortas from PP2A^SMKO^ mice, while the secretory markers such as mmp2, mmp9, col1a1 and col3a1 were increased (Figure 3F). Those results suggested that PP2Acα deficiency may lead to VSMC dedifferentiation.

### 2.4 PP2Acα Maintains the Contractile Phenotype of VSMC

**Figure 4:**
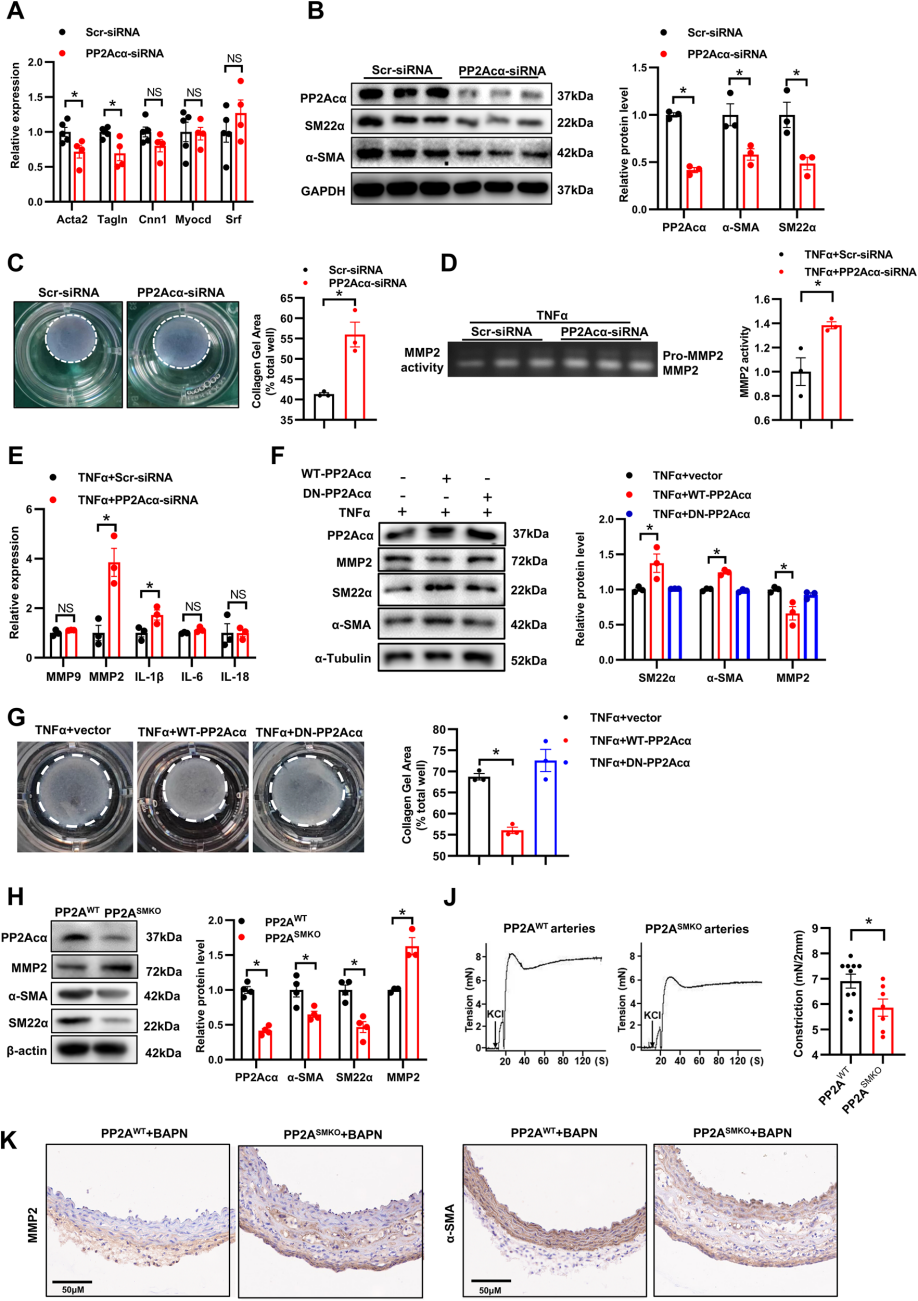
PP2Acα maintains the contractile phenotype of VSMCs. A) VSMCs were transfected with the Scr‐siRNA or PP2Acα‐siRNA (50 nM) for 24 h. The quantitative analysis of mRNA levels of acta2, tagln, cnn1, myocardin, and srf. *N* = 3, Student's *t* test. B) VSMCs were transfected with the Scr‐siRNA or PP2Acα‐siRNA (50 nM). After 24 h, the cells were serum‐starved for 24 h. The representative Western blot images and quantitative analysis of protein levels of PP2Acα, α‐SMA, and SM22α. *N* = 3, Student's *t* test. C) Collagen‐based contraction assay and quantitative analysis in VSMCs transfected with the Scr‐siRNA or PP2Acα‐siRNA (50 nm). *N* = 3, Student's *t* test. D) VSMCs were transfected with the Scr‐siRNA or PP2Acα‐siRNA (50 nM). After 24 h, the cells were serum‐starved for 48 h. Gelatin zymogram showed the activity of MMP2 and its quantitative result. *N* = 3, Student's *t* test. E) VSMCs were transfected with the Scr‐siRNA or PP2Acα‐siRNA (50 nM) for 24 h, then were treated with TNFα (20 µg/mL) for 24 h. The quantitative analysis of mRNA levels of MMP9, MMP2, IL‐1β, IL‐6, and IL‐18. *N* = 3, Student's *t* test. F) VSMCs were transfected with WT‐PP2Acα or dominant negative PP2Acα (DN‐PP2Acα) plasmid for 48 h and then treated with TNFα (20 µg/mL) for 24 h. Representative Western blot images and quantification analysis of α‐SMA, SM22α, and MMP2. *N* = 3, one‐way ANOVA followed by the Tukey's test. G) Collagen‐based contraction assay and quantitative analysis in VSMCs treated with TNFα and transfected with WT‐PP2Acα or DN‐PP2Acα plasmid. *N* = 3, one‐way ANOVA followed by the Tukey's test. H) The representative Western blot images and quantitative analysis of protein levels of PP2Acα, α‐SMA, SM22α, and MMP2 in the aortas from PP2A^WT^ and PP2A^SMKO^ mice. *N* = 3‐4, Student's *t* test. J) Contraction force of mesenteric arteries from the PP2A^WT^ and PP2A^SMKO^ mice in the stimulation of 100 mmol L^−1^ KCl. *N* = 7‐10, Student's *t* test. K) Immunohistochemical staining of α‐SMA or MMP2 in aortic sections from the BAPN‐treated PP2A^WT^ and PP2A^SMKO^ mice. ^*^ *P* < 0.05. NS means no significant difference.

Next, we verified whether PP2Acα is required for the maintenance of the contractile phenotype of VSMCs. Our result showed that PP2Acα silencing reduced VSMC contractile phenotype markers (α‐SMA and SM22α) mRNA expression and protein levels (**Figure** **4**A,B). In accordance, PP2Acα silencing reduced VSMC contractility, as indicated by a collagen gel contraction assay (Figure 4C). PP2Acα‐silenced VSMCs exhibited greater MMP2 secretion induced by TNFα, as indicated by gelatin‐zymography (Figure 4D). Additionally, PP2Acα deficiency resulted in a further elevation of VSMC synthetic markers, specifically MMP2 and IL1β, following TNFα stimulation (Figure 4E). Similarly, PDGF‐BB‐stimulated expression of EREG and SPP1 was augmented in PP2Acα‐deficient VSMCs (Figure S6, Supporting Information). In contrast, PP2Acα overexpression attenuated TNFα‐driven phenotypic switching, as demonstrated by preserved contractile marker expression and gel contraction capacity (Figure 4F,G). However, the overexpression of PP2Acα‐L199P, a dominant‐negative mutant form of PP2Acα, did not preserve contractile phenotype, suggesting the requirement of PP2A enzymatic activity for this regulatory function. DT061 (PP2A agonist) reversed TNFα‐mediated downregulation of contractile proteins, validating the critical role of PP2A activity (Figure S7, Supporting Information). Altogether, these results revealed that PP2A maintains VSMC contractile phenotype, while PP2Acα deficiency promotes VSMC conversion to the synthetic phenotype.

A concordant pattern was also observed in PP2A^WT^ and PP2A^SMKO^ mouse aortas. The aortas from the PP2A^SMKO^ mice exhibited reduced α‐SMA and SM22α protein levels, but increased MMP2 protein levels (Figure 4H). Mesenteric arteries from PP2A^SMKO^ mice showed a reduced response to KCl, suggesting decreased vascular smooth muscle contractility in the PP2A^SMKO^ mice (Figure 4J). In addition, immunohistochemistry confirmed that the PP2A^SMKO^ mouse aortas had higher expression of MMP2 and lower expression of α‐SMA when compared with the PP2A^WT^ in AAD (Figure 4K). Collectively, these results suggest that PP2Acα deficiency‐induced VSMC dedifferentiation may lead to an accelerated AAD disease progression.

### 2.5 PP2Acα Deficiency Induces VSMC Phenotypic Switching in a KLF4‐Dependent Manner

**Figure 5:**
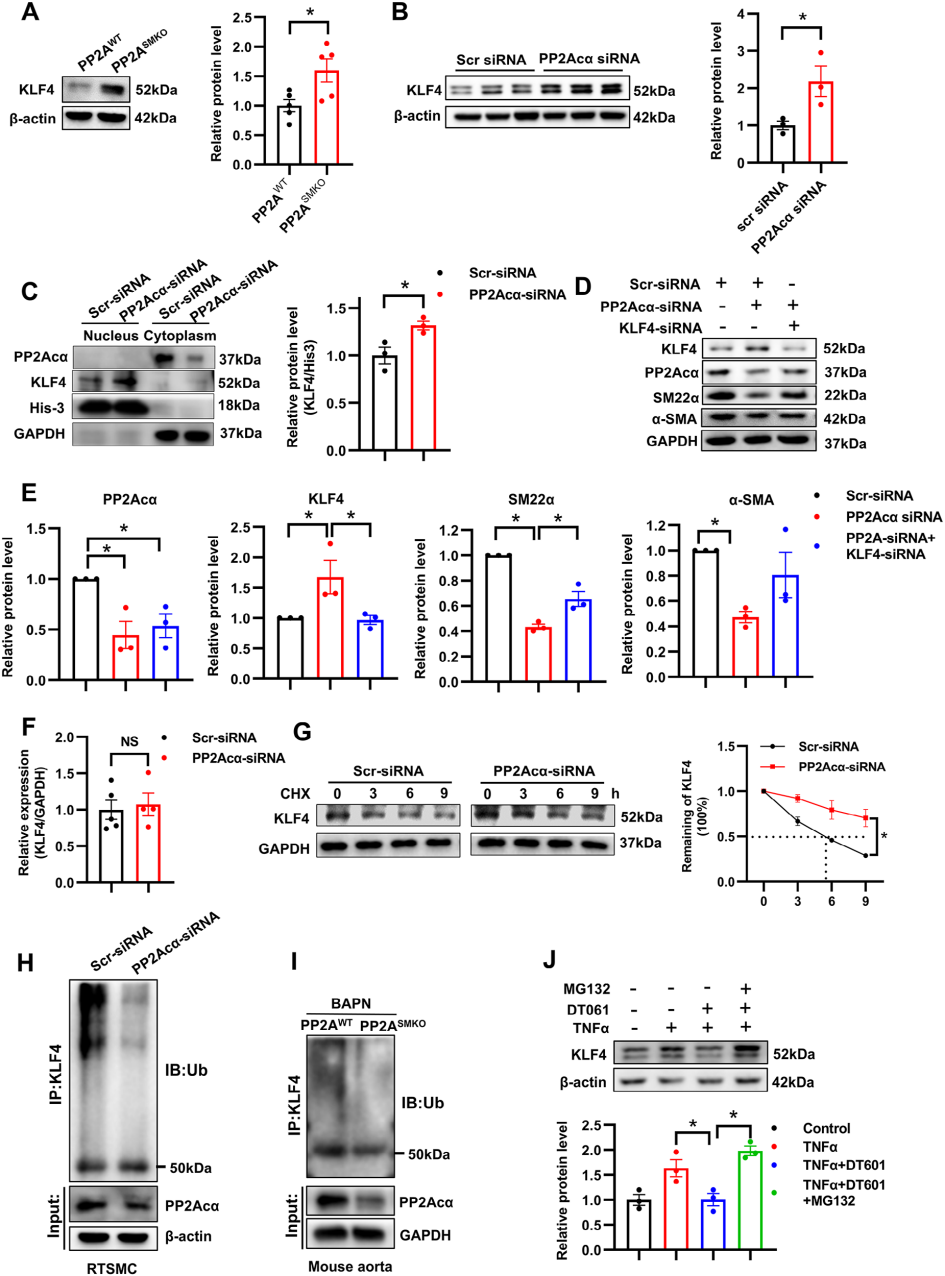
PP2Acα deficiency triggers VSMC differentiation by stabilizing KLF4. A) Representative Western blot images and quantification analysis of KLF4 in the aortas from PP2A^WT^ and PP2A^SMKO^ mice. *N* = 5, Student's *t* test. B) Representative Western blot images and quantification analysis of KLF4 in the VSMCs transfected with Scr‐siRNA or PP2Acα‐siRNA (50 nM). *N* = 5, Student's *t* test. C) Representative Western blot images and quantification of KLF4 in nuclear and cytoplasm extracts from the VSMCs transfected with Scr‐siRNA or PP2Acα‐siRNA (50 nM). *N* = 3, Student's *t* test. VSMCs were transfected with PP2Acα‐siRNA (50 nM) either in the presence or absence of KLF4‐siRNA (50 nM), followed by serum deprivation for 24 h. D) Representative Western blot images and E) quantitative analysis of PP2Acα, KLF4, α‐SMA, and SM22α. *N* = 3, one‐way ANOVA followed by Tukey's test. G) VSMCs were transfected with the Scr‐siRNA or PP2Acα‐siRNA (50 nM). After 48 h, VSMCs were treated with cycloheximide (CHX, 20 µg/mL) for 0, 3, 6, and 9 h. Representative Western blot images and quantification analysis of KLF4. *N* = 3, two‐way ANOVA. H) VSMCs were transfected with the Scr‐siRNA or PP2Acα‐siRNA. After 48 h, VSMCs were treated with MG132 (10 µm) for 9 h. A representative blot of KLF4 ubiquitination is shown, *N* = 3. I) Aortic tissues from the BAPN‐treated PP2A^WT^ and PP2A^SMKO^ mice. A representative blot of KLF4 ubiquitination is shown, *N* = 3. J) VSMCs were pre‐treated with PP2A agonist DT061 (10 µM) for 1 h and then exposed to TNFα (20 µg/mL) stimulation for 24 h in the presence or absence of MG132 (10 µM). Representative Western blot images and quantification analysis of KLF4. *N* = 3, one‐way ANOVA followed by the Tukey's test. ^*^ *P* < 0.05.

Myocardin/SRF (serum response factor) complex formation is the molecular basis of sustaining VSMC contractile genes transcription and contractile phenotypic state. Upon pathological stimulation, the upregulated VSMC reprogramming factor KLF4 (Kruppel‐like factor 4) disrupts the myocardin/SRF complex and then leads to VSMC phenotypic switching.^[^ ^20^ ^]^ Since our previous results showed that the absence of PP2Acα does not influence the expression of myocardin and SRF (Figure 3F), we tried to investigate whether PP2Acα modulated VSMC phenotypes through regulating KLF4. Consistent with the previous reports,^[^ ^21^ ^]^ our results demonstrate a significant increase in KLF4 expression in human AAD tissues (Figure S8A, Supporting Information). We then examined if the level of KLF4 was impacted by PP2Acα and found that PP2Acα deficiency in vitro significantly upregulated KLF4 protein level (**Figure** **5**A). The upregulated KLF4 protein level was further observed in the PP2A^SMKO^ mouse aortas (Figure 5B). Moreover, nuclear protein was extracted for analysis of KLF4 protein expression and the results showed that KLF4 levels in the nuclei increased in the PP2Acα‐knockdown VSMCs (Figure 5C), indicating a role for KLF4 in the PP2Acα‐modulated VSMC phenotypes. Notably, the reduced contractile markers induced by PP2Acα knockdown were also nullified after KLF4 silencing (Figure 5D,E). These results demonstrate that the trigger of VSMC phenotypic switching by PP2Acα deficiency is KLF4‐dependent.

As PP2Acα deficiency had no effects on the mRNA level of KLF4 in VSMCs (Figure 5F), we tested whether PP2Acα exerted a post‐translational effect on KLF4 protein stability, and the result showed that PP2Acα deficiency increased the half‐life of KLF4 protein in VSMCs (Figure 5G). The ubiquitin‐proteasome pathway has been proven to play an important role in KLF4 protein quality control.^[^ ^22^ ^]^ Therefore, we proposed that PP2Acα may regulate KLF4 protein stability by modulating its ubiquitination. In support of this hypothesis, knockdown of PP2Acα in VSMCs led to a decrease in KLF4 ubiquitination (Figure 5H). Furthermore, in vivo analysis revealed a reduction in KLF4 ubiquitination in the aortas of PP2A^SMKO^ mice compared to PP2A^WT^ mice treated with BAPN (Figure 5I). DT061 reduced the elevated KLF4 protein level induced by TNFα, the effect of which was abolished by the proteasome inhibitor MG132 (Figure 5J). These results suggest that PP2Acα deficiency elevates KLF4 protein levels by suppressing its ubiquitination‐dependent degradation.

### 2.6 PP2Acα Deficiency Increases KLF4 Phosphorylation through Activating AKT1

**Figure 6:**
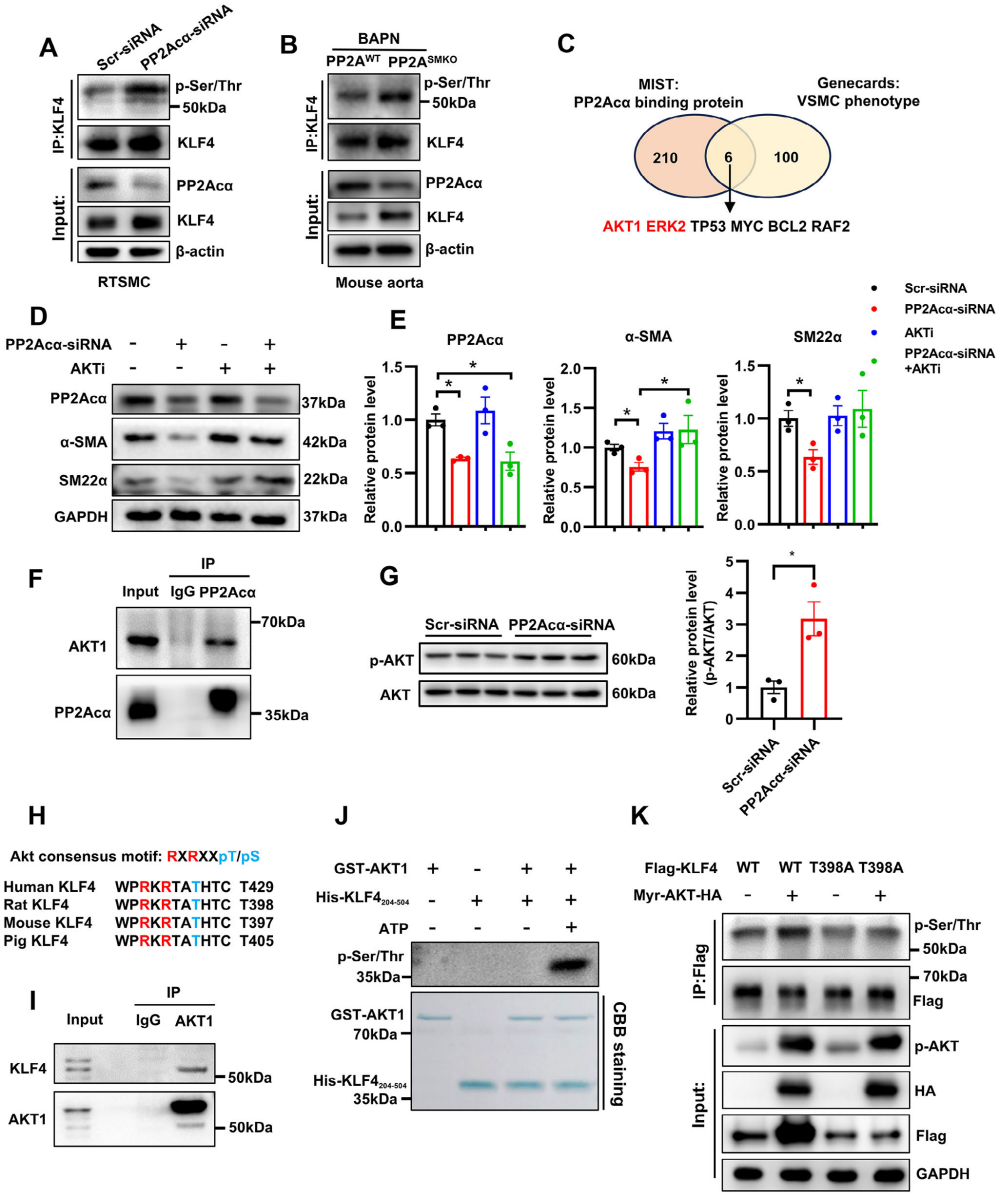
Increased KLF4 phosphorylation due to PP2Acα deficiency is mediated by AKT1. Immunoblotting using phospho‐Thr/Ser‐specific antibody to detect phosphorylated KLF4 level after immunoprecipitation with KLF4 from A) VSMCs transfected with Scr‐siRNA or PP2Acα‐siRNA and B) Aortic tissues of the BAPN‐treated PP2A^WT^ and PP2A^SMKO^ mice. A representative blot is shown, *N* = 3. C) The overlapping of PP2Acα‐binding proteins with VSMC phenotype‐related proteins. D) Representative Western blot images and E) quantification of PP2Acα, α‐SMA, and SM22α in the VSMCs transfected with PP2Acα‐siRNA (50 nM) for 6 h and then treated with AKTi (AKT inhibitor, MK2206, 5 µM) for 48 h. *N* = 3, two‐way ANOVA followed by the Bonferroni test. F) Endogenous coimmunoprecipitation assay between PP2Acα and AKT1 in VSMCs using an anti‐PP2Acα antibody or control IgG. A representative blot is shown, *N* = 3. G) Representative Western blot images and quantification of p‐AKT in the VSMCs transfected with PP2Acα‐siRNA (50 nM) for 48 h. *N* = 3, Student's *t* test. H) The predicted AKT phosphorylation sites in KLF4 are highly conserved during evolution. Putative AKT phosphorylation sites are indicated in blue. I) Endogenous coimmunoprecipitation assay between KLF4 and AKT1 in VSMCs using an anti‐AKT1 antibody or control IgG. A representative blot is shown, *N* = 3. J) Recombinant GST‐AKT1 and His‐KLF4_204‐504_ were co‐incubated without or with 500 µM ATP in a kinase buffer. The Ser/Thr‐phosphorylation of KLF4 was detected by phospho‐Thr/Ser antibody, and the protein loading was verified by Coomassie brilliant blue (CBB) staining. *N* = 3. K) FLAG‐tagged WT KLF4 or Thr398 mutant (T398A) KLF4 and HA‐tagged activated AKT (myr‐AKT‐HA) were transfected into HEK‐293T cells. Immunoblotting using phospho‐Thr/Ser‐specific antibody to detect phosphorylated KLF4 levels after immunoprecipitation of FLAG from HEK‐293T cells. A representative blot is shown, *N* = 3. ^*^ *P* < 0.05.

Considerable evidence indicates that KLF4 ubiquitination is modulated by its phosphorylation status.^[^ ^23^, ^24^, ^25^ ^]^ Notably, our findings reveal an elevation in the total serine/threonine phosphorylation level of KLF4 within human AAD tissues (Figure S8B, Supporting Information). Considering that PP2Acα depends on its phosphatase activity to preserve the contractile phenotype of VSMCs, we propose a potential mechanism wherein PP2Acα modulates KLF4 ubiquitination by influencing its phosphorylation status. To confirm our speculation, we first detected KLF4 phosphorylation level after PP2Acα knockdown. Compared with control, PP2Acα deficiency increased serine/threonine phosphorylation level of KLF4, as detected by immunoblotting with anti‐phospho‐serine/threonine antibody (**Figure** **6**A). In vivo analysis also demonstrated that VSMCs PP2Acα deficiency elevated KLF4 phosphorylation level in the aortas of BAPN‐treated mice (Figure 6B). Our results showed that KLF4 was mainly located in the nucleus, while PP2Acα was mainly located in the cytoplasm. Besides, PP2Acα also did not bind to endogenous KLF4 in VSMCs (Figure S9A, Supporting Information), indicating that KLF4 phosphorylation was indirectly regulated by PP2A. Therefore, we proceeded to define the substrate for PP2A dephosphorylation substrate by which PP2A indirectly regulates the phosphorylation of KLF4. The online tool MIST was used to screen out PP2Acα‐binding proteins and a total of 210 proteins were identified. Through further overlapping with the Genecards database (top 100 VSMCs phenotype‐related genes), six known proteins involved in the regulation of VSMC phenotype were identified as potential PP2A substrates (Figure 6C). Among these, AKT1 and extracellular regulated protein kinase 2(ERK2) were the only two kinase/phosphatase and more likely mediate the regulatory effect of PP2A on KLF4 phosphorylation. Besides, AKT1 and ERK2 have previously been confirmed to be the substrates for PP2A dephosphorylation.^[^ ^26^, ^27^ ^]^ So, we next validated whether PP2A maintains the contractile phenotype of VSMCs by directly dephosphorylating AKT1 or ERK2. It showed that AKT inhibitor, but not ERK inhibitor, prevented the decreased contractile marker protein level induced by PP2Acα deficiency in VSMCs (Figure 6D,E; Figure S9B, Supporting Information). Consistent with the previous report, the binding between endogenous PP2Acα and AKT was confirmed in VSMCs (Figure 6F), and PP2Acα deficiency also resulted in increased phosphorylation of AKT both in vivo and in vitro (Figure 6G; Figure S9C, Supporting Information). These results suggest that AKT may be the substrate for dephosphorylation by PP2A and mediates the maintenance effect of PP2A on VSMC contractile phenotype.

Then, we examined whether the increased phosphorylation of KLF4 in the PP2Acα‐knockdown VSMCs is also mediated by AKT. Scansite identified the Ser478 site of KLF4 as a likely site for AKT phosphorylation based on the consensus AKT phosphorylation target motif X‐R‐X‐X‐S/T (Figure 6H). We first confirmed that AKT1 could bind to KLF4 in VSMCs (Figure 6I). An in vitro immunoprecipitation assay using full‐length and truncation mutants of Flag‐KLF4 indicates that the C‐terminal domain of KLF4 interacts with AKT1 (Figure S10, Supporting Information). Furthermore, an in vitro kinase assay was employed to examine the phosphorylation of KLF4 by AKT1. Recombinant KLF4_204‐504_ were incubated with activated recombinant AKT1 in the presence of ATP, and the result showed that AKT1 directly phosphorylated KLF4 (Figure 6J). Then, HEK 293T cells were transfected with plasmids encoding Myr‐AKT‐HA, Flag‐KLF4 WT, or Flag‐KLF4 T398A (mutating Thr398), and the results showed that the activated AKT could increase the phosphorylation of KLF4, while mutating Thr398 (T398A) reduced AKT‐induced KLF4 phosphorylation (Figure 6K). Overall, these results suggest that PP2Aca deficiency leads to increased AKT1 phosphorylation and kinase activity, thereby increasing AKT1 substrate KLF4 phosphorylation at the Thr398 site.

### 2.7 PP2Acα Deficiency Induces Phosphorylation‐Dependent Inhibition of Ubiquitination and Degradation of KLF4 through Activating AKT

**Figure 7:**
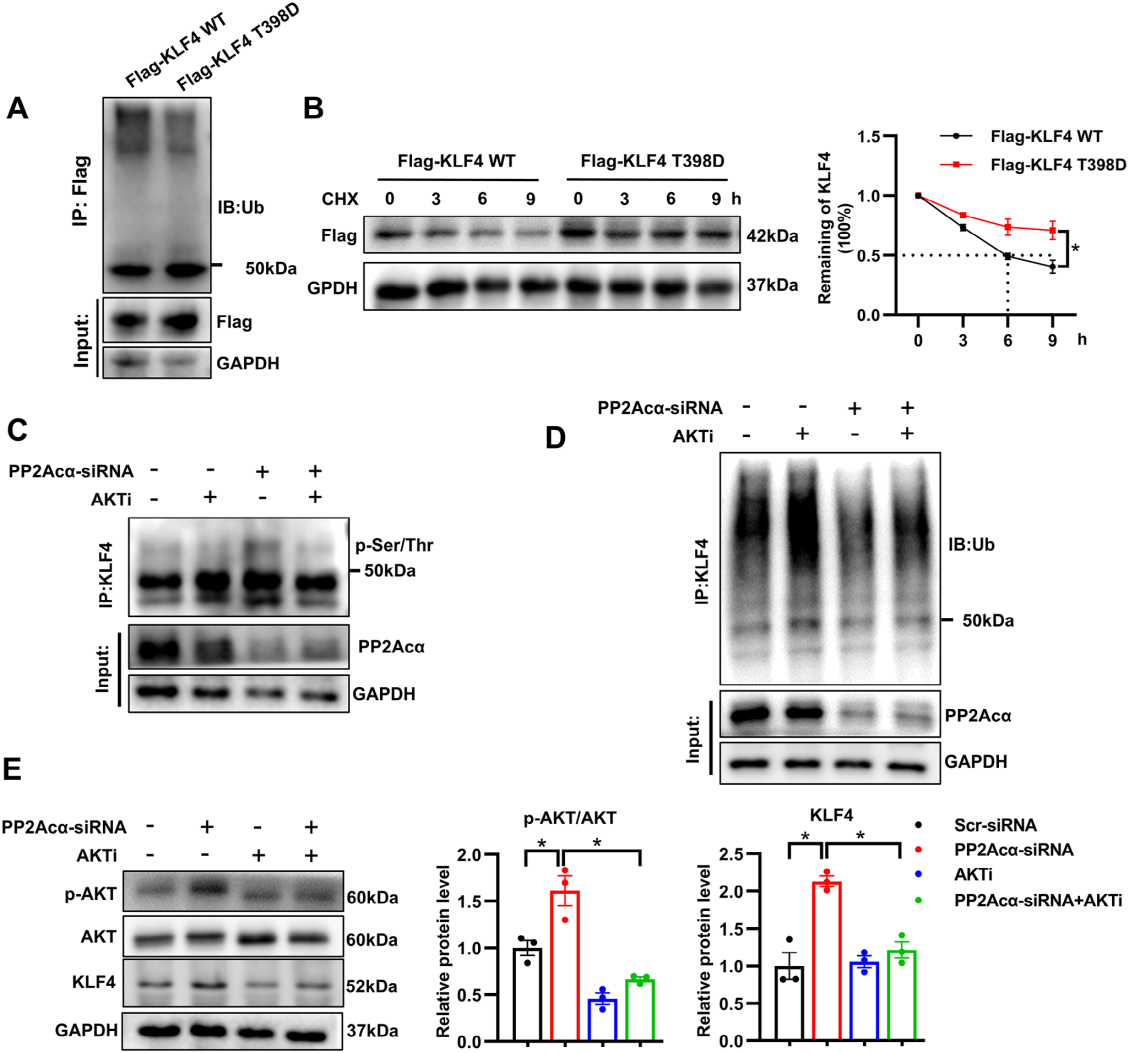
PP2Acα deficiency induces phosphorylation‐dependent inhibition of ubiquitination and degradation of KLF4 by activating AKT. A) VSMCs were transfected with KLF4 WT or T389D plasmids. After 48 h, VSMCs were treated with MG132 (10 µM) for 9 h. Representative Western blot image of FLAG‐tagged KLF4 ubiquitination, *N* = 3. B) Representative Western blot images and quantification analysis of KLF4 in VSMCs transfected with FLAG‐tagged WT KLF4 or Thr398 mutant (T398D) KLF4 for 48 h and then treated with CHX (20 µg/mL) for 0, 3, 6, and 9 h. *N* = 3, two‐way ANOVA. C) VSMCs were transfected with PP2Acα‐siRNA (50 nM) for 6 h and then treated with or without AKTi (AKT inhibitor, MK2206, 5 µM) for 48 h, and immunoblotting using phospho‐Thr/Ser specific antibody to detect phosphorylated KLF4 level after immunoprecipitation of KLF4 from VSMCs. A representative blot is shown, *N* = 3. D) VSMCs were transfected with PP2Acα‐siRNA (50 nM) for 6 h and then treated with or without AKTi (AKT inhibitor, MK2206, 5 µM). After 48 h, VSMCs were treated with MG132 (10 µM) for 9 h. Representative Western blot image of KLF4 ubiquitination, *N* = 3. E) Representative Western blot images and quantification analysis of p‐AKT, MMP2, and KLF4 in VSMCs transfected with PP2Acα‐siRNA (50 nM) for 6 h and treated with AKTi (AKT inhibitor, MK2206, 5 µM) for 48 h. *N* = 3, two‐way ANOVA followed by Bonferroni test. ^*^ *P* < 0.05.

To determine whether the phosphorylation of the Thr398 site affects the protein stability of KLF4, plasmids were constructed with a phosphomimetic mutation of KLF4 (KLF4‐T398D). Compared with KLF4‐WT, KLF4‐T398D plasmid transfection decreased the ubiquitination of KLF4 and increased KLF4 protein half‐life (**Figure** **7**A,B). This suggests that T398 is a critical phosphorylation site that contributes to the stabilization of KLF4 by AKT1. More importantly, PP2Acα deficiency‐induced up‐regulation of KLF4 phosphorylationwere alleviated by AKT inhibitor (Figure 7C). To investigate whether other kinases also contribute to the elevated KLF4 phosphorylation resulting from PP2Acα depletion, we treated PP2Acα‐deficient VSMCs with mammalian target of rapamycin (mTOR) and protein kinase c (PKC) inhibitors. Results demonstrated that neither inhibitor altered the elevated KLF4 phosphorylation following PP2Acα depletion, confirming exclusive regulation of KLF4 phosphorylation through AKT1 (Figure S11, Supporting Information). Besides, AKT inhibitor suppressed PP2Acα deficiency‐induced reduction in KLF4 ubiquitination and concomitantly normalized the elevated KLF4 protein accumulation in VSMCs (Figure 7D,E). In general, these results indicate that PP2Acα deficiency‐induced Akt phosphorylation results in phosphorylation of AKT1 substrate KLF4 at T398, which blocks KLF4 ubiquitination‐dependent degradation and eventually leads to VSMC dedifferentiation, resulting in an aggravated AAD.

### 2.8 PP2Acα Overexpression in Abdominal Aortas Rescues AAA in PP2A^SMKO^ Mice

**Figure 8:**
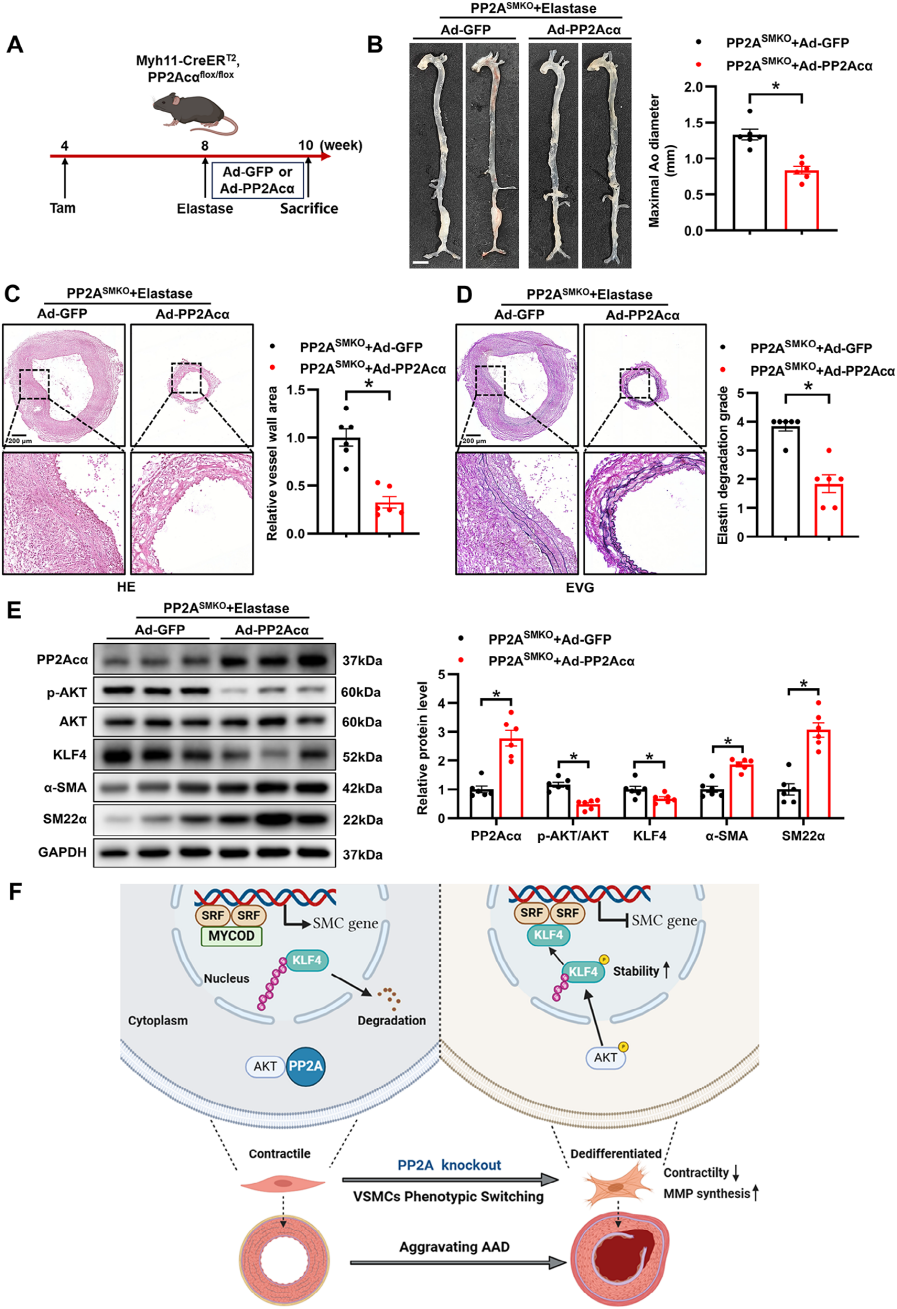
PP2Acα overexpression rescues AAA in PP2A^SMKO^ mice. A) Experimental design. After elastase injury, eight‐week‐old PP2A^SMKO^ mice were subsequently infected with adenovirus control (Ad‐GFP) or PP2Acα‐expressing adenovirus (Ad‐PP2Acα) through pluronic F127 hydrogel delivery. B) Representative aorta ultrasound images and maximal abdominal aortic diameters of PP2A^SMKO^ mice after treatment as described in A. Scale bars = 2.5 mm. Student's *t* test. C) Representative images of HE staining and quantification of vessel wall area of PP2A^SMKO^ mice in A. D) Representative images of EVG staining and quantification of the elastin degradation grade of PP2A^SMKO^ mice in A. Scale bars = 200 µm. Mann–Whitney test. E) Representative Western blot images and quantification analysis of PP2Acα, p‐AKT, KLF4, α‐SMA and SM22α in the abdominal aortas of PP2A^SMKO^ mice in A. Student's *t* test F) Graphic summary of the PP2A/AKT1/KLF4 axis in VSMC phenotype conversion and AAD development. ^*^ *P* < 0.05.

Given that PP2Acα deficiency in VSMCs exacerbates the progression of AAA, we sought to rescue PP2A^SMKO^ mice from AAA development through PP2Acα overexpression. To achieve PP2Acα overexpression, adenovirus carrying PP2Acα (Ad‐PP2Acα) or GFP plasmid (Ad‐GFP) was locally delivered by pluronic‐F127 hydrogel to the infrarenal abdominal aortas of elastase‐treated PP2A^SMKO^ mice to achieve PP2Acα overexpression (**Figure** **8**A). PP2Acα overexpression protected the elastase‐induced dilation of the abdominal aortas in PP2A^SMKO^ mice (Figure 8B). Elastase‐induced thickening of the vascular wall and elastin degradation in the aortas of PP2A^SMKO^ mice were also evidently prevented by PP2Acα overexpression (Figure 8C,D). Western blot analysis confirmed that Ad‐PP2Acα delivery successfully achieved PP2Acα overexpression in the abdominal aortas of PP2A^SMKO^ mice (Figure 8E). Furthermore, PP2Acα overexpression upregulated the expression of α‐SMA and SM22α, while concurrently downregulating KLF4 and p‐AKT levels in the elastase‐treated PP2A^SMKO^ mouse abdominal aortas (Figure 8E). These results were consistent with the in vitro findings, demonstrating that PP2Acα deficiency induces VSMCs phenotypic switching through activation of the AKT1‐KLF4 signaling axis (Figure 8F). Collectively, these findings indicate that restoration of PP2Acα expression prevents VSMCs phenotypic switching and AAA in murine models.

## 3 Discussion

In this study, we observed that PP2Acα protein level in the aortas was decreased both in human and mouse AAD samples. Deficiency of PP2Acα in VSMCs aggravates the development and progression of BAPN and elastase‐induced murine AAD. Moreover, PP2Acα deficiency causes VSMC phenotype switching from contractile to synthetic phenotype, which is accompanied by decreased contractility and increased MMP2 secretion. Mechanistically, PP2Acα deficiency induces phosphorylation‐dependent inhibition of KLF4 proteasomal degradation through activating AKT1, and thus increases KLF4 protein level and inhibits the transcription of VSMC contractile genes. Our results highlight the importance of PP2A‐AKT‐KLF4 signaling axis in regulating VSMC phenotypic switch and AAD progression.

PP2A is one of the most abundant phosphatases and is essential for the regulation of intracellular signaling. By dephosphorylating proteins involved in the signaling pathway, PP2A can lead to signaling activation or termination of signals that have been generated.^[^ ^28^ ^]^ Different from the previous knowledge in phosphatases, PP2A has excellent substrate specificity by binding to different regulatory subunits. PP2A dysregulation has been reported to be closely associated with several diseases, including lung fibrosis, cardiac hypertrophy, kidney injury, and cancer.^[^ ^13^, ^15^, ^16^, ^29^ ^]^ Due to the PP2Acα subunit performing the catalytic activity of the PP2A holoenzyme, changes in PP2Acα expression, phosphorylation or methylation are the main mechanisms of the regulation of PP2A activity. Previous studies have found that PP2A activity is decreased in human aortic aneurysmal samples and the PP2A inhibitor accelerated Ang II‐induced AAA progression.^[^ ^30^ ^]^ Herein, we found that PP2Acα is enriched in VSMCs compared to other cell populations in the aortic wall. Besides, PP2Acα expression is decreased in mouse and human AAD tissues, particularly within VSMCs. The transcriptional regulation of PP2Acα expression involves intricate epigenetic mechanisms. Specifically, prior research has demonstrated that histone deacetylase 1 (HDAC1) is recruited to the first intron region of the PP2Acα gene via the transcription factor Ikaros, where it mediates chromatin deacetylation and facilitates a condensed chromatin conformation, thereby suppressing PP2Acα transcriptional activity.^[^ ^31^ ^]^ Notably, Lin et al. reported that HDAC1 expression is upregulated in VSMCs of AAD tissues and contributes to VSMC phenotypic switching.^[^ ^32^ ^]^ Building on these findings, we speculate that aberrant HDAC1 activation in AAD‐associated VSMCs may epigenetically repress PP2Acα transcription through chromatin remodeling. This proposed mechanism warrants further investigation to elucidate the interplay between HDAC1 dysregulation, PP2Acα downregulation, and pathological vascular remodeling in AAD pathogenesis. A key finding of this study is the mechanistic demonstration that VSMC‐specific PP2Acα deficiency exacerbates both BAPN‐induced AAD and elastase‐triggered AAA, supporting the potential therapeutic application of PP2A agonists in AAD. It is worth noting that PP2A is a potential target for anti‐tumor therapy^[^ ^33^ ^]^; PP2A inhibitors exhibit remarkable anti‐tumor effects in the ongoing preclinical and clinical studies (NCT06012734, NCT06065462, NCT05809830). Our study suggests that the adverse effects of PP2A inhibitors on the cardiovascular system should be noted.

VSMC phenotypic switch from contractile/differentiated to synthetic/dedifferentiated phenotype was known to be the key cellular process in the cardiovascular diseases associated with vascular remodeling. In AAD pathogenesis, reduced contractility in medial synthetic VSMCs, partly owing to a downregulated expression of the contractile proteins, limits extracellular matrix contraction and then promotes the formation of intramural edema, which is a key step in the formation of AAD. Moreover, synthetic VSMCs secrete more MMPs and inflammatory factors, leading to matrix degradation of the vessel walls. Importantly, previous research has reported that mutations of the VSMC contractile phenotype‐related genes (Talgn, Acta2) caused the formation of AAD. Besides, the inhibition of IL6 or MMP2 prevented the formation of Ang II‐induced mouse AAD. Herein, we found that PP2Acα deficiency results in the loss of VSMC contractile protein and contractility and exacerbated MMP2 expression both in vitro and in vivo. Furthermore, ablation of PP2Acα exacerbates BAPN‐induced reductions in VSMC contractile proteins and accelerates ECM degradation. Collectively, these findings implicate PP2Acα deficiency‐driven VSMC phenotypic switching as a critical determinant in the pathogenesis of AAD progression. It is noteworthy that emerging evidence highlights the pleiotropic actions of PP2Acα within diverse vascular wall cell lineages. For example, macrophage‐specific deletion of PP2Acα reduces inflammatory cytokine secretion and macrophage infiltration in kidney fibrosis models,^[^ ^34^ ^]^ suggesting a potential immunomodulatory function in vascular inflammatory processes. Furthermore, pharmacological inhibition of PP2A using LB100 disrupts endothelial barrier integrity, a critical early event in the pathogenesis of AAD.^[^ ^35^ ^]^ Collectively, these findings prompt critical inquiry into the cell‐type‐specific roles of PP2Acα in AAD. Moving forward, dissecting the heterogeneous functions of PP2Acα across distinct cellular compartments of AAD represents a pivotal frontier for unlocking the full therapeutic promise of targeting this phosphatase in AAD management.

Next, we explored the mechanism of PP2Acα deficiency‐mediated VSMC phenotypic switching. KLF4, a member of the Kruppel‐like transcription factor family, is one of the most important differentiation repressors and is crucial in VSMC reprogramming and aneurysm development.^[^ ^20^, ^21^ ^]^ By inhibiting the formation of SRF‐MYOCD transcription complexes and directly binding to the G/C repressor, induction of KLF4 in VSMCs results in a decrease of the VSMC differentiation makers.^[^ ^36^ ^]^ KLF4 is also thought to maintain the transcription of VSMC dedifferentiation genes as a transcription activator.^[^ ^20^, ^37^ ^]^ As presented here, PP2Acα deficiency led to a rise in KLF4 protein level, but not in mRNA. KLF4 knockdown attenuated VSMC dedifferentiation caused by PP2A deficiency. Due to the short half‐life of KLF4, ubiquitination‐dependent protein degradation is critical to its protein level.^[^ ^38^ ^]^ We found that PP2Acα deficiency decreased KLF4 ubiquitination and extended its half‐life in VSMCs. Correspondingly, the suppressing effect of PP2A agonists on KLF4 protein level was abolished by the proteasome inhibitor MG132. These findings indicate that PP2Acα regulates VSMC differentiation by promoting KLF4 ubiquitination.

Interestingly, recent studies found that KLF4 ubiquitination is regulated by its phosphorylation state. For example, phosphorylation of KLF4 at Ser234 increases KLF4 K63‐linked ubiquitination and protein stability,^[^ ^25^ ^]^ while KLF4 Ser123 phosphorylation upregulates KLF4 poly ubiquitination and degradation.^[^ ^24^ ^]^ Given that PP2Acα maintains the VSMC contractile phenotype depends on its phosphatase activity, we speculated that PP2Acα may regulate KLF4 phosphorylation, thus inhibiting its ubiquitination and protein stability. Our study revealed that PP2Acα deficiency increased Ser/Thr phosphorylation of KLF4. However, there does not exist an interaction between PP2Acα and KLF4, suggesting that PP2Acα indirectly regulates KLF4 phosphorylation. By analyzing the PP2A‐binding proteins that have been reported and VSMC phenotype‐related proteins, AKT1 is regarded as a prospective substrate for PP2A in the regulation of VSMC phenotype. Numerous studies have demonstrated that PP2A directly dephosphorylates AKT1 at Ser473, thus inhibiting its kinase activity.^[^ ^39^ ^]^ It has been reported that AKT activation is associated with reduced ubiquitination of KLF4.^[^ ^40^ ^]^ Consistent with a previous study,^[^ ^41^ ^]^ we found that AKT1 phosphorylates KLF4 at Thr398. Further results suggest that KLF4 phosphorylation at the T398 site inhibits its ubiquitination and degradation. Moreover, AKT inhibitor alleviates VSMC dedifferentiation and KLF4 upregulation caused by PP2A knockdown. It is known that phosphorylation of AKT1 increases in the human AAA tissues,^[^ ^42^ ^]^ the factors that result in AKT inhibition have been demonstrated to suppress the formation of experimental aortic aneurysm.^[^ ^43^, ^44^ ^]^ Here, we reveal for the first time that the upregulated AKT phosphorylation in aneurysm tissues may be due to the reduction of PP2Acα, the activated AKT causes phosphorylation‐dependent inhibition of ubiquitination and degradation of KLF4, ultimately leading to VSMC dedifferentiation. Notably, the kinase regulatory network in VSMCs within the aneurysm pathological microenvironment exhibits remarkable complexity. Beyond the PP2A/AKT1 pathway, kinases such as mothers against decapentaplegic homolog 3 (SMAD3), ERK1/2, and polo‐like kinase 1 (PLK1) are also activated under aneurysm pathological stimuli,^[^ ^45^, ^46^, ^47^ ^]^ and prior studies have shown that: SMAD3 can inhibit KLF4 ubiquitination and degradation by inducing phosphorylation at the Ser254 residue^[^ ^23^ ^]^; ERK1/2‐mediated phosphorylation at Ser123 promotes KLF4 degradation^[^ ^24^ ^]^; and PLK1‐mediated modification at Ser234 exerts a protective effect by suppressing degradation.^[^ ^25^ ^]^ These findings suggest that distinct phosphorylation sites on KLF4 may differentially regulate its stability and influence the progression of AAD. Future studies are needed to further elucidate the predominant forms of KLF4 phosphorylation that exert regulatory roles in AAD, as well as whether cross‐talk exists among different kinase signals to integrate the regulation of KLF4 phosphorylation and protein stability.

Although our study provides new insights into the role of PP2A in VSMC phenotypes and AAD, there are some limitations here. First, it remains unclear how the phosphorylation of KLF4 at Thr398 affects its ubiquitination. We speculate that KLF4 Thr398 phosphorylation may affect the interaction of the responsible ubiquitin ligases or deubiquitylates with KLF4. In addition, it is needed to use KLF4 VSMC‐specific knockdown mice to clarify in vivo that PP2A deficiency exacerbates VSMC dedifferentiation and AAD is mediated by KLF4. Finally, the molecular mechanisms by which PP2A expression is downregulated in the AAD and dedifferentiated VSMCs need to be further elucidated.

Overall, our study reveals an important role for PP2A in maintaining VSMC contractile phenotype and inhibiting AAD. Mechanistically, VSMC‐specific PP2Acα deficiency accelerates AAD pathogenesis through AKT1‐dependent phosphorylation and stabilization of KLF4, which orchestrates VSMC phenotypic switch. The translational relevance of this work is substantiated by concordant downregulation of PP2Acα and upregulation of phosphorylated KLF4 in human AAD samples, positioning the PP2A‐AKT1‐KLF4 signaling axis as a critical determinant in AAD pathobiology.

## 4 Experimental Section

### Animals

Animal research was conducted in accordance with the guidelines of the Animal Care and was approved by the Biomedical Ethics Committee of Peking University (DLASBD0070). Healthy male C57BL/6J mice (8‐10 weeks, 20–25 g) were provided by the Department of Laboratory Animal Science, Peking University Health Science Center (Beijing, China). The *Myh11‐CreER^T2^.PPP2ca^flox/flox^* mice were kindly provided by Prof. Yang Chen from Guangzhou Medical University. Briefly, male *Myh11‐CreER^T2^.PPP2ca^flox/flox^* mice were treated with tamoxifen at 75 mg kg^−1^ for 5 consecutive days at the age of three‐weeks‐old and were referred to as PP2A^SMKO^ mice; The same mice treated with corn oil were referred to as PP2A^WT^ mice. The primer used for genotyping were: *PPP2ca^flox/flox^* forward primer: 3′‐TAGCCCATGCCTTTAATCTCAGAGC‐5′, reverse: 3′‐CACTCGTCGTAGAACCCATAAACC‐5′; *Myh11‐CreER^T2^* forward primer: 3′‐CAGCCAACTTTACGCCTAGC‐5′, reverse: 3′‐TCTCAAGATGGACCTAATACGG‐5′. All mice were exposed to a 12 h‐light/dark cycle under defined environmental conditions at 25 ± 2 °C with a relative humidity of 50% and were given free access to food and water.

### Animal Models—BAPN‐Induced AAD Model

Five‐week‐old male PP2A^WT^ and PP2A^SMKO^ mice were fed with water containing 0.5% β‐aminopropionitrile (BAPN, Sigma) for 4 weeks. Moreover, to evaluate the therapeutic potential of PP2A as an intervening target in AAD, three‐week‐old male C57BL/6J mice were fed with water containing 0.4% BAPN.^[^ ^48^ ^]^ After 7 days of BAPN treatment, DT061 (PP2A agonist) was injected intraperitoneally (3 mg kg^−1^ day^−1^), continuing throughout the remainder of the experiment for 14 days in total.

### Animal Models—PPE‐Induced AAA Mouse Model

Eight‐ to ten‐week‐old male PP2A^WT^ and PP2A^SMKO^ mice were anesthetized with an intraperitoneal injection (i.p.) of sodium pentobarbital (50 mg kg^−1^), and the mouse abdominal aortas from the infra‐renal aorta to the bifurcation of the aorta were isolated by blunt dissection. Each separated abdominal aorta was then wrapped circumferentially with blotting (bibulous) paper soaked with 1.5 U PPE (Sigma) for 50 min, and mice in the saline group were treated with saline. After which, the blotting paper was removed from the abdominal aorta and the abdomen was sutured up.

At the end of the experiment, mice were anesthetized with sodium pentobarbital (100 mg kg^−1^), perfused from the heart and fixed with 4% paraformaldehyde. Perivascular connective tissues were removed from the entire aortas under a dissecting microscope and photographed by an independent investigator, and the maximum aortic diameters were analyzed and calculated separately using Image J software.

### Cell Isolation and Culture

As described previously,^[^ ^49^ ^]^ the isolation and subsequent culture of primary VSMCs from the aortas of Sprague‐Dawley rats (150 g) was achieved through the collagenase digestion method, as described previously. The experiments were conducted using primary VSMCs in passages 3 to 9. HEK293T cell line was purchased from the American Type Culture Collection (ATCC). All cells were maintained in high glucose Dulbecco's minimum essential medium supplemented with 10% FBS, 100 U/mL penicillin, and 100 µg/mL streptomycin at 37 °C in a humidified atmosphere with 5% CO_2_.

### Histological Analysis

The collected aortas were fixed in paraffin, then the peritubular connective tissues were removed, embedded in OCT, and sliced into 5 µm thick sections. Hematoxylin‐eosin (HE) and Verhoeff's Van Gieson (EVG) staining were performed for the valuation of vessel wall injury and elastin degradation. Elastin degradation was graded as follows: < 25%, 1 point; 25–50%, 2 points; 50–75%, 3 points; > 75%, 4 points.

### Human Aortic Samples

The use of human aortic samples in this study was approved by the ethics committee of Peking University People's Hospital (2023PHB170‐001), with all participants or legally designated representatives of organ donors having written informed consent. For Western blot analysis, aortic tissue samples were obtained from five patients undergoing Stanford type A aortic dissection repair surgeries at Peking University People's Hospital. During surgical intervention, the full‐thickness segments of the ascending aorta were promptly excised and fresh tissues were immediately procured. Control samples comprised full‐thickness ascending aortic tissues from five non‐AAD cases. Following excision, both AAD and non‐AAD aortic specimens were preserved in ice‐cold physiological saline solution, after which periaortic adipose tissues and mural thrombotic deposits were meticulously dissected away. The aortic tissues were then rapidly frozen in liquid nitrogen and stored at −80 °C until subsequent protein extraction procedures. The detailed information of all patients is listed in Table S1 (Supporting Information).

### Immunohistochemistry and Immunofluorescence

Sections were placed in a 60 °C oven for 1 h and then deparaffinized with xylene. Antigen extraction was stored in citrate buffer (pH 6.0) at 95 °C for 60 min.

For immunohistochemistry, after prehybridization and inactivation of endogenous peroxidative activity using a 3% H_2_O_2_ solution, sections were blocked with 5% goat serum for 1 h at 37 °C and then incubated with primary antibodies overnight at 4 °C. Sections were then incubated with the HRP‐conjugated secondary antibodies at 37 °C for 60 min. Hematoxylin and diaminobenzidine (DAB) were used to visualize nuclei and antibody deposition, respectively. All photomicrographs were taken with PRECICE 510A fully automatic digital slice (UNIC, China).

For immunofluorescence, sections were blocked with 5% goat serum for 1 h at 37 °C and then incubated with primary antibodies overnight at 4 °C, followed by secondary DyLight 488 or 555‐conjugated antibody for 1 h at room temperature. Subsequently, coverslips were mounted using DAPI‐containing antifade mounting medium. Images were captured with a fluorescence microscopy (Nikon, Japan).

### RNA Sequencing

After obtaining the aortic tissues from PP2A^WT^ and PP2A^SMKO^ mice, the samples were sent to NovelBio Corp. Laboratory in Shanghai for transcriptome sequencing. The RNA extraction was carried out using TRIzol reagent (Invitrogen). RNA quality assessments, including spectrophotometry and agarose gel electrophoresis, were performed to determine the 18S and 28S rRNA ratio. RNA seq sequencing was performed on the BGI‐500 sequencing platform. The raw sequencing reads were filtered and processed by HISAT software to reference the genome, and RSEM software was used to quantify gene expression. Differently expressed genes (DEGs) were screened by DEG seq. Genes with |log2Foldchange|>0.5 and FDR(q‐value) < 0.05 between groups were considered significantly expressed ones. Finally, functional enrichment analysis was performed by the DAVID online software and the Gene Set Enrichment Analysis (GSEA).

### Plasmid Preparation

The plasmids pcDNA3.1/PP2Acα‐WT, pT3/myr‐AKT‐HA were purchased from Miaolingbio (Suzhou, China). The plasmid FLAG‐KLF4‐WT was kindly provided by Prof. Li‐Long Pan from Jiangnan University. To prepare PP2Acα serial deletion mutant constructs, cDNA sequences corresponding to different regions were amplified by PCR from the PP2Acα‐WT construct, and subcloned into the pcDNA3.1 (+) vector. For the generation of individual plasmids encoding KLF4‐T398D (replacement of threonine 398 by asparagine, T398D), KLF4‐T398A (replacement of threonine 398 by alanine, T398A), and DN‐PP2A‐L199P (replacement of leucine 199 by proline, L199P), one step cloning kit was used (Vazyme, China).

### Plasmid and Small Interfering RNA (siRNA) Transfection

Plasmid transfections were performed with NanoTrans Transfection Reagent (CYTOCH, China) according to the manufacturer's instructions. The siRNAs against rat PP2Acα and KLF4, as well as scrambled siRNA, were designed and synthesized by GenePharma (China). Sequences for PP2Acα siRNA, 5′‐GCCGUGACCACUUUAGAAUTT‐3′; sequences for KLF4 siRNA,5′‐ ACCUUGCCUUACACAUGAATT‐3′. The siRNA transfections were performed using Lipofectamine RNAiMax Reagent (Invitrogen, USA) according to the manufacturer's instructions.

### Western Blot

Cells or tissues were lysed with ice‐cold RIPA buffer (Applygen, China) containing phenylmethylsulfonyl fluoride (1 mM). Samples were centrifuged at 13 000 rpm for 15 min at 4 °C, the supernatant was then collected, and protein concentration was determined using a BCA kit (Thermo Scientific, USA). Equal amounts of proteins were separated by 8–12% SDS‐PAGE gels and then transferred to a 0.22 µm nitrocellulose membrane. After incubating with 5% bovine serum albumin (BSA, Solarbio, China), membranes were incubated with primary antibodies at 4 °C overnight, followed by secondary antibodies (ZSBIO, China) for 1.5 h at room temperature. Subsequently, the bands were visualized by enhanced chemiluminescence reaction (ECL, Applygen, China). The grayscale of the blots was analyzed using NIH Image J software.

### qRT‐PCR

RNA was extracted from either tissues or cells using the Trizol reagent (Transgen, China), then RNA was reverse‐transcribed using a reverse transcription kit (Transgen, China). SYBR Green 2×PCR Mix (Transgen, China) was used to determine mRNA expression. The expression was calculated using the ^ΔΔ^CT method with GAPDH as an internal reference. The primer sequences for target genes are listed in Table S1 (Supporting Information).

### Blood Pressure Measurement

Basal blood pressure values of PP2A^WT^ and PP2A^SMKO^ mice were measured using a noninvasive tail‐cuff blood pressure measurement system (Kent Scientific Corporation). The equipment was placed in a clean and quiet environment free from odors and noise, and the mice were tested for 7 consecutive days of training from 1 pm to 5 pm daily to acclimatize them to the system. A single investigator then performed basal blood pressure measurements at the same time for 3 consecutive days. Ten recorded measurements were taken for each mouse each day, and the average of 30 measurements was calculated as the blood pressure of each mouse.

### Gelatin Zymography

Gelatin zymography experiments were performed as described previously.^[^ ^50^ ^]^ Briefly, cells were transfected with siRNA or plasmid in a serum‐free DMEM medium. After 48 h, the supernatant of the culture medium was collected and mixed with a non‐reducing sample buffer. Samples were then electrophoresed on SDS‐PAGE gels containing 7.5% gelatin. SDS was removed from the gel with 2.5% Triton X‐100, and the gel was incubated in an incubation buffer for 24 h at 37 °C. The gels were stained with 0.5% coomassie brilliant blue staining solution and then decolored with decolorizing solution (50% ddH_2_O, 40% methanol, 10% acetic acid) to assess the activity of MMPs.

### Collagen Gel Contraction Assay

The contractility of rat VSMCs was measured using a cell contraction assay kit (cell Biolabs, USA) according to the manufacturer's protocol. After transfection of siRNA or plasmid for 48 h, cells were harvested and resuspended in DMEM containing 10% FBS at a density of 5 × 10^6^ cells/mL and mixed with ice‐cold collagen gel solution at a volume ratio of 1:4. Cell‐collagen mixture with a volume of 500 µL was added to a 24‐well plate and incubated at 37 °C for 30 min. Then, 1 mL DMEM containing 10% FBS was added to the collagen gel lattice. After 24 h, the gel was photographed and analyzed for statistics using Image J software.

### Wire Myography

Myography was performed as described previously.^[^ ^51^ ^]^ Briefly, mesenteric arteries of PP2A^WT^ and PP2A^SMKO^ mice were isolated and placed in ice‐cold Krebs‐Henseleit buffer. After the removal of perivascular connective tissue, the mesenteric artery was cut into rings (2 mm) and mounted to myograph chambers (Danish Myo Technology, Denmark) to measure vascular tension. After 30 min of equilibration at 37 °C, mesenteric arteries were tested with KCl (80 mmol L^−1^) to assess vascular reactivity and then flushed. The responses to KCl (100 mmol L^−1^) were obtained to detect vasocontraction.

### Co‐IP

Cells and aortas were lysed with NP40 (Keygentec, China) on ice for 30 min and centrifuged at 12 000 rpm at 4 °C for 15 min. Then, the supernatant was taken, the protein concentration was measured using the BCA method, and the input sample was left. The supernatant was incubated overnight at 4 °C with the primary antibody, and then pre‐washed protein A/G plus‐agarose (Santa Cruz, USA) was added to the samples and incubated for 5 h at 4 °C. After centrifuging at 2000 rpm 4 °C for 3 min, immunoprecipitates were washed three times with IP Lysis Buffer. Then, immunoprecipitates were mixed with the loading Buffer and boiled for 5 min. Finally, the samples were tested by Western blot.

### Adenovirus Infection of Mouse Abdominal Aortas

The adenovirus for mouse PP2Acα (Ad‐PP2Acα) and GFP (Ad‐GFP) were purchased from Hanheng Biotechnology (China). For adenovirus infection of the abdominal aortas in mice,^[^ ^52^ ^]^ the mouse abdomen was opened, and the infrarenal abdominal aorta was isolated. Followed by elastase injury, 40 µL of 22.5% w/v pluronic F‐127 gel containing 1.2 × 10^9^ plaque‐forming units (pfu) of Ad‐GFP or Ad‐PP2Acα was placed around the infrarenal abdominal aorta.

### Single‐Cell RNA‐seq Analysis from Public Datasets

Single‐cell RNA sequencing (scRNA‐seq) data obtained from the aortas of mice with abdominal aortic aneurysm (AAA) were downloaded from the GEO database using accession number GSE118237. Both the count matrix and normalized expression matrix were processed using the R package “Seurat”. All datasets were merged, normalized, scaled, clustered, and visualized using t‐distributed stochastic neighbor embedding (t‐SNE). Marker genes for each cluster were identified using the FindAllMarkers function with default parameters, applying thresholds of *p* < 0.05 and log2 fold change > 0.1. VSMCs were stratified into PPP2CA+ and PPP2CA− clusters based on PPP2CA expression. PPP2CA+ VSMCs were defined as having more than 0 reads in the PPP2CA gene locus. Conversely, VSMCs with 0 reads mapping to PPP2CA were classified as PPP2CA−.

### In Vitro Kinase Assay

Recombinant human GST‐AKT1 (100 ng) and human His‐KLF4_204‐504_ (300 ng) were incubated in 40 µL kinase buffer (50 mM Tris‐HCl/pH 7.5, 50 mM MgCl_2_, 1 mM sodium orthovanadate, 1 mM DTT, 100 mM KCl) containing 500 µM ATP at 37 °C for 1 h. The reaction was terminated by heating the samples in protein loading buffer. The phosphorylation of KLF4 was assessed by Western blot.

### Statistical Analysis

All experiments were repeated independently at least three times using vessels and cells from different animals. Data were presented as the means ± standard errors of the means (SEM). The statistical analyses were performed using GraphPad Prism 8.0 software (USA). For 2‐group comparisons of normally distributed data, Student's *t* test was applied. For comparisons among three or more groups, one‐way ANOVA followed by Tukey's test was employed. Two‐way ANOVA followed by Bonferroni test analysis was used when >2 groups and variables were compared. Survival curves were evaluated with the Kaplan–Meier method and compared by log‐rank tests. AAD incidence was evaluated with the Chi‐square test. Elastin degradation scores were analyzed by the Mann–Whitney test. In all cases, a statistically significant difference was present when the probability was less than 0.05. The detailed statistical analysis applied to each experiment is presented in the corresponding figure legends.

## Conflict of Interest

The authors declare no conflict of interest.

## Author Contributions

W.‐P.H. contributed to methodology, investigation, resources, data analysis, writing of the original draft, visualization, writing, reviewing, and editing. Z.‐Y.C. was involved in methodology, investigation, resources, data analysis, and writing of the original draft. Q.‐L.L. contributed to the conceptualization and funding acquisition. T.Z. participated in writing, reviewing, and editing. X.‐Y.C. and C.W. were responsible for the investigation. Q.‐Y.Z. provided technical support. R.Q. was involved in project administration, supervision, writing, reviewing, editing, and funding acquisition.

## Supporting information



Supporting Information

## Data Availability

The data that support the findings of this study are available from the corresponding author upon reasonable request.

## References

[1] J. Gao , H. Cao , G. Hu , Y. Wu , Y. Xu , H. Cui , L. HS , L. Zheng , Signal Transduct. Target Ther. 2023, 8, 55. 36737432 10.1038/s41392-023-01325-7 PMC9898314

[2] J. Golledge , S. Thanigaimani , J. T. Powell , P. S. Tsao , Eur. Heart J. 2023, 44, 2682. 37387260 10.1093/eurheartj/ehad386 PMC10393073

[3] K. B. Rombouts , T. A. R. van Merrienboer , J. C. F. Ket , N. Bogunovic , J. van der Velden , K. K. Yeung , Eur. J. Clin. Invest. 2022, 52, 13697. 10.1111/eci.13697 PMC9285394 34698377

[4] K. B. Rombouts , T. A. R. van Merrienboer , J. C. F. Ket , N. Bogunovic , J. van der Velden , K. K. Yeung , Eur. J. Clin. Invest. 2022, 52, 13697. 10.1111/eci.13697 PMC9285394 34698377

[5] G. K. Owens , M. S. Kumar , B. R. Wamhoff , Physiol. Rev. 2004, 84, 767. 15269336 10.1152/physrev.00041.2003

[6] T. T. Zhang , Q. Q. Lei , J. He , X. Guan , X. Zhang , Y. Huang , Z. Y. Zhou , R. X. Fan , T. Wang , C. X. Li , J. Y. Shang , Z. M. Lin , W. L. Peng , L. K. Xia , Y. L. He , C. Y. Hong , J. S. Ou , R. P. Pang , X. P. Fan , H. Huang , J. G. Zhou , Circulation 2023, 148, 589. 37203562 10.1161/CIRCULATIONAHA.122.063029

[7] M. Ding , Y. Xie , R. J. Wagner , Y. Jin , A. C. Carrao , L. S. Liu , A. K. Guzman , R. J. Powell , J. Hwa , E. M. Rzucidlo , K. A. Martin , Arterioscler. Thromb. Vasc. Biol. 2011, 31, 1403. 21454807 10.1161/ATVBAHA.110.216804 PMC3100723

[8] Z. Li , W. Kong , Cell Signal. 2020, 70, 109575. 32088371 10.1016/j.cellsig.2020.109575

[9] F. Zhang , X. Guo , Y. Xia , L. Mao , Cell. Mol. Life Sci. 2021, 79, 6. 34936041 10.1007/s00018-021-04079-z PMC11072026

[10] C. M. O'Connor , A. Perl , D. Leonard , J. Sangodkar , G. Narla , Int. J. Biochem. Cell Biol. 2018, 96, 182. 29107183 10.1016/j.biocel.2017.10.008 PMC5927617

[11] C. Lambrecht , D. Haesen , W. Sents , E. Ivanova , V. Janssens , Methods Mol. Biol. 2013, 1053, 283. 23860660 10.1007/978-1-62703-562-0_17

[12] A. R. Clark , M. Ohlmeyer , Pharmacol. Ther. 2019, 201, 181. 31158394 10.1016/j.pharmthera.2019.05.016 PMC6700395

[13] M. Gu , M. Tan , L. Zhou , X. Sun , Q. Lu , M. Wang , H. Jiang , Y. Liang , Q. Hou , X. Xue , Z. Xu , C. Dai , Kidney Int. 2022, 102, 321. 35483524 10.1016/j.kint.2022.03.024

[14] D. L. Brautigan , C. Farrington , G. Narla , Clin. Sci. 2021, 135, 1545. 10.1042/CS20201367 PMC9059670 34192314

[15] S. Chen , L. Chen , L. Ye , Y. Jiang , Q. Li , H. Zhang , R. Zhang , H. Li , D. Yu , R. Zhang , Y. Niu , Q. Zhao , J. Liu , G. Ouyang , M. Aschner , Y. Zheng , L. Zhang , W. Chen , D. Li , J. Hazard. Mater. 2022, 424, 127624. 34740159 10.1016/j.jhazmat.2021.127624

[16] L. Li , C. Fang , D. Xu , Y. Xu , H. Fu , J. Li , Am. J. Transl. Res. 2016, 8, 1769. 27186301 PMC4859906

[17] M. Ehling , W. Celus , R. Martín‐Pérez , R. Alba‐Rovira , S. Willox , D. Ponti , M. C. Cid , E. A. V. Jones , G. Di Conza , M. Mazzone , Circ. Res. 2020, 127, 707. 32527198 10.1161/CIRCRESAHA.119.316071 PMC7616433

[18] J. D. Stone , A. Narine , P. R. Shaver , J. C. Fox , J. R. Vuncannon , T. DA , Am. J. Physiol. Heart Circ. Physiol. 2013, 304, H369. 23203966 10.1152/ajpheart.00446.2012 PMC3774500

[19] K. I. Jeon , H. Jono , C. L. Miller , Y. Cai , S. Lim , X. Liu , P. Gao , J. Abe , J. D. Li , C. Yan , FEBS J. 2010, 277, 5026. 21078118 10.1111/j.1742-4658.2010.07908.x PMC3059601

[20] C. Yap , A. Mieremet , C. J. M. de Vries , D. Micha , V. de Waard , Arterioscler. Thromb. Vasc. Biol. 2021, 41, 2693. 34470477 10.1161/ATVBAHA.121.316600 PMC8545254

[21] M. Salmon , W. F. Johnston , A. Woo , N. H. Pope , G. Su , G. R. Upchurch Jr. , G. K. Owens , G. Ailawadi , Circulation 2013, 128, S163. 24030402 10.1161/CIRCULATIONAHA.112.000238 PMC3922284

[22] N. K. Dhaliwal , K. Miri , S. Davidson , H. Tamim El Jarkass , J. A. Mitchell , Stem Cell Rep. 2018, 10, 1308. 10.1016/j.stemcr.2018.02.007 PMC6000723 29526737

[23] C. Lei , H. Kan , X. Xian , W. Chen , W. Xiang , X. Song , J. Wu , D. Yang , Y. Zheng , Nat. Commun. 2023, 14, 5360. 37660071 10.1038/s41467-023-41177-x PMC10475135

[24] M. O. Kim , S. H. Kim , Y. Y. Cho , J. Nadas , C. H. Jeong , K. Yao , D. J. Kim , D. H. Yu , Y. S. Keum , K. Y. Lee , Z. Huang , A. M. Bode , Z. Dong , Nat. Struct. Mol. Biol. 2012, 19, 283. 22307056 10.1038/nsmb.2217

[25] J. Mai , Z. Y. Zhong , G. F. Guo , X. X. Chen , Y. Q. Xiang , X. Li , H. L. Zhang , Y. H. Chen , X. L. Xu , R. Y. Wu , Y. Yu , Z. L. Li , X. D. Peng , Y. Huang , L. H. Zhou , G. K. Feng , X. Guo , R. Deng , X. F. Zhu , Theranostics 2019, 9, 3541. 31281496 10.7150/thno.32908 PMC6587166

[26] G. Nadel , Z. Yao , E. Wainstein , I. Cohen , I. Ben‐Ami , A. Schajnovitz , G. Maik‐Rachline , Z. Naor , B. A. Horwitz , R. Seger , Cell Commun. Signal. 2022, 20, 5. 34998390 10.1186/s12964-021-00805-z PMC8742922

[27] H. Chung , D. L. Brautigan , Cell Signal. 1999, 11, 575. 10433518 10.1016/s0898-6568(99)00033-9

[28] R. Baskaran , B. K. Velmurugan , Life Sci. 2018, 210, 40. 30170071 10.1016/j.lfs.2018.08.063

[29] E. R. Lubbers , P. J. Mohler , J. Mol. Cell. Cardiol. 2016, 101, 127. 27832939 10.1016/j.yjmcc.2016.11.003 PMC5939568

[30] X. Zhou , C. Zhang , F. Xie , W. Wei , R. Li , Q. Xu , Y. Wang , P. A. Klenotic , G. Narla , N. Dong , Z. Lin , Clin. Sci. 2021, 135, 2085. 10.1042/CS20210315 PMC8612712 34402501

[31] K. Nagpal , K. S. Watanabe , B. P. Tsao , G. C. Tsokos , J. Biol. Chem. 2014, 289, 13751. 24692537 10.1074/jbc.M114.558197 PMC4022849

[32] L. Sun , C. Wang , Y. Yuan , Z. Guo , Y. He , W. Ma , J. Zhang , J. Cell. Physiol. 2020, 235, 8747. 32324261 10.1002/jcp.29718

[33] D. Perrotti , P. Neviani , Lancet Oncol. 2013, 14, 229. 10.1016/S1470-2045(12)70558-2 PMC3913484 23639323

[34] L. Sun , E. M. Hult , T. T. Cornell , K. K. Kim , T. P. Shanley , C. A. Wilke , M. Agarwal , S. J. Gurczynski , M. BB , M. K. Dahmer , Am. J. Physiol. Lung Cell Mol. Physiol. 2019, 316, L1035. 30838865 10.1152/ajplung.00299.2018 PMC6620666

[35] X. L. Bai , Q. Zhang , L. Y. Ye , Q. D. Hu , Q. H. Fu , X. Zhi , W. Su , R. G. Su , T. Ma , W. Chen , S. Z. Xie , C. L. Chen , T. B. Liang , Mol. Cancer Ther. 2014, 13, 2062. 24867249 10.1158/1535-7163.MCT-13-0800

[36] D. Gomez , G. K. Owens , Cardiovasc. Res. 2012, 95, 156. 22406749 10.1093/cvr/cvs115 PMC3388816

[37] P. Y. Chen , L. Qin , G. Li , J. Malagon‐Lopez , Z. Wang , S. Bergaya , S. Gujja , A. W. Caulk , S. I. Murtada , X. Zhang , Z. W. Zhuang , D. A. Rao , G. Wang , Z. Tobiasova , B. Jiang , R. R. Montgomery , L. Sun , H. Sun , E. A. Fisher , J. R. Gulcher , C. Fernandez‐Hernando , J. D. Humphrey , G. Tellides , T. W. Chittenden , M. Simons , Cell Stem Cell. 2020, 26, 542. 32243809 10.1016/j.stem.2020.02.013 PMC7182079

[38] D. Hu , M. Gur , Z. Zhou , A. Gamper , M. C. Hung , N. Fujita , L. Lan , I. Bahar , Y. Wan , Nat. Commun. 2015, 6, 8419. 26420673 10.1038/ncomms9419 PMC4598737

[39] D. M. Virshup , Curr. Opin. Cell Biol. 2000, 12, 180. 10712915 10.1016/s0955-0674(99)00074-5

[40] Y. Sun , B. Zheng , X. H. Zhang , M. He , Z. W. Guo , J. K. Wen , Biochem. Biophys. Res. Commun. 2014, 443, 382. 24321547 10.1016/j.bbrc.2013.11.129

[41] X. Dai , P. Liu , A. W. Lau , Y. Liu , H. Inuzuka , Cancer Med. 2014, 3, 1211. 25116380 10.1002/cam4.298 PMC4302671

[42] A. Ghosh , G. Lu , G. Su , B. McEvoy , O. Sadiq , P. D. DiMusto , A. Laser , J. S. Futchko , P. K. Henke , J. L. Eliason , G. R. Upchurch Jr. , Am J. Pathol. 2014, 184, 148. 24332015 10.1016/j.ajpath.2013.09.016 PMC3873487

[43] R. Liu , J. Huang , Y. Ge , S. Liu , T. Huang , H. Cai , B. Pan , Q. Zhang , P. Yang , M. Liao , B. Xu , W. Wang , Eur. J. Vasc. Endovasc. Surg. 2020, 60, 254. 32423743 10.1016/j.ejvs.2020.03.042

[44] M. J. Ruiz‐Rodríguez , J. Oller , S. Martínez‐Martínez , I. Alarcón‐Ruiz , M. Toral , Y. Sun , Á. Colmenar , M. J. Méndez‐Olivares , D. López‐Maderuelo , C. B. Kern , J. F. Nistal , A. Evangelista , G. Teixido‐Tura , M. R. Campanero , J. M. Redondo , EMBO Mol. Med. 2024, 16, 132. 38177536 10.1038/s44321-023-00009-7 PMC10897446

[45] L. Y. Kong , C. Liang , P. C. Li , Y. W. Zhang , S. D. Feng , D. H. Zhang , R. Yao , L. L. Yang , Z. Y. Hao , H. Zhang , X. X. Tian , C. R. Guo , B. B. Du , J. Z. Dong , Y. Z. Zhang , J. Am. Heart Assoc. 2022, 11, 026174. 10.1161/JAHA.122.026174 PMC9673629 36314496

[46] J. Gong , D. Zhou , L. Jiang , P. Qiu , D. M. Milewicz , Y. E. Chen , B. Yang , Arterioscler. Thromb. Vasc. Biol. 2020, 40, 1651. 32404006 10.1161/ATVBAHA.120.313033 PMC7316596

[47] X. Da , Z. Li , X. Huang , Z. He , Y. Yu , T. Tian , C. Xu , Y. Yao , Q. K. Wang , Nat. Commun. 2023, 14, 2265. 37081014 10.1038/s41467-023-37809-x PMC10119315

[48] X. Yang , C. Xu , F. Yao , Q. Ding , H. Liu , C. Luo , D. Wang , J. Huang , Z. Li , Y. Shen , W. Yang , Z. Li , F. Yu , Y. Fu , L. Wang , Q. Ma , J. Zhu , F. Xu , X. Cong , W. Kong , Eur. Heart J. 2023, 44, 1248. 36638776 10.1093/eurheartj/ehac823

[49] Y. Wang , C. Chen , Q. Wang , Y. Cao , L. Xu , R. Qi , Br. J. Pharmacol. 2019, 176, 282. 30302749 10.1111/bph.14515 PMC6295405

[50] Q. Zhang , Z. Cai , Z. Yu , C. Di , Y. Qiu , R. Qi , Cardiovasc. Drugs Ther. 2023, 39, 239. 37979015 10.1007/s10557-023-07518-0

[51] Z. Cai , N. Xie , Z. Gong , Z. Yang , F. Lin , Z. Li , R. Dai , Y. Chen , S. Zhang , S. Zhu , S. Zhou , J. Lin , F. Yu , L. Liu , J. Sun , J. Zhou , W. Li , C. Xiong , Y. Fu , X. Cong , W. Kong , Hypertension 2023, 80, 1231. 36999441 10.1161/HYPERTENSIONAHA.122.20654

[52] G. Zhao , Y. Zhao , H. Lu , Z. Chang , H. Liu , H. Wang , W. Liang , Y. Liu , T. Zhu , O. Rom , Y. Guo , L. Chang , B. Yang , M. T. Garcia‐Barrio , J. D. Lin , Y. E. Chen , J. Zhang , J. Clin. Invest. 2022, 132. 10.1172/JCI158309 PMC9621131 36066968

