# Supplementary material for: PP2Acα Deficiency in Vascular Smooth Muscle Cells Accelerates Aortic Aneurysm and Dissection by Regulating KLF4 Phosphorylation and Ubiquitination
Source: Adv Sci (Weinh). 2025 Jul 28;12(37):e00102. doi: 10.1002/advs.202500102 (PMC12499488; doi:10.1002/advs.202500102)
Supplement: Supplementary file 1 — Supporting Information [file ADVS-12-e00102-s001.docx]

**ONLINE SUPPLEMENTAL DOCUMENT**

**PP2Acα Deficiency in Vascular Smooth Muscle Cells Accelerates Aortic Aneurysm and Dissection by Regulating** **KLF4 Phosphorylation and Ubiquitination**

by

Wei-Peng Hu^1,2,3,4,5^, Ze-Yu Cai^2,6^, Qing-Le Li^7^, Tao Zhang^7^, Xiang-Yu Chu^7^, Chang Wang^1,2,3,4,5^, Qing-Yi Zhang^1,2,3,4,5^, Rong Qi^1,2,3,4,5,^*

1. Department of Pharmacology, School of Basic Medical Sciences, Peking University Health Science Center, 38 Xueyuan Road, Haidian District, Beijing 100191, China.

2. State Key Laboratory of Vascular Homeostasis and Remodeling, Peking University, Beijing 100191, China.

3. State Key Laboratory of Natural and Biomimetic Drugs, Peking University, Beijing 100191, China.

4. NHC Key Laboratory of Cardiovascular Molecular Biology and Regulatory Peptides, Peking University, Beijing 100191, China.

5. Beijing Key Laboratory of Molecular Pharmaceutics and New Drug Delivery Systems, Peking University, Beijing 100191, China.

6. Department of Physiology and Pathophysiology, School of Basic Medical Sciences, Peking University Health Science Center, Beijing 100191, China.

7. Department of Vascular Surgery, Peking University People's Hospital, Beijing 100044, China.

***Corresponding Author:**

Rong Qi, email: [ronaqi@bjmu.edu.cn](mailto:ronaqi@bjmu.edu.cn), ORCID: 0000-0002-0627-9968

**Table S1. Patient information**

|  | non-AAD1 | non-AAD2 | non-AAD3 | non-AAD4 | non-AAD5 | AAD1 | AAD2 | AAD3 | AAD4 | AAD5 |
| --- | --- | --- | --- | --- | --- | --- | --- | --- | --- | --- |
| Age | 46 | 51 | 44 | 41 | 55 | 44 | 36 | 44 | 56 | 41 |
| Gender | Male | Male | Male | Male | Male | Male | Male | Male | Male | Male |
| Coronary heart disease | No | No | No | No | No | No | No | No | No | No |
| Hyperlipidemia | No | Yes | Yes | No | No | No | Yes | No | Yes | No |
| Diabetes | No | No | No | No | No | No | Yes | No | Yes | No |
| Smoking | No | No | No | Yes | No | No | No | No | No | No |

**Table S2. A list of primers used for RT-PCR.**

| **Target** | **Forward** **primer (5’-3’)** | **Reverse primer (5’-3’)** |
| --- | --- | --- |
| Rat-acta2 | ACTCTGGAGATGGCGTGACTC | GCGTTCATTCCCGATGGT |
| Rat-calponin | CCAGCATGTCTTCCGCACACT | CCATGAAGTTGCTCCCGATG |
| Rat-tagln | TCCTTCCAGCCCACAAACGAC | GGGCCACACTGCATTACAATC |
| Rat-SRF | TAGACACGAAACAGGCTCAGG | GGCATCGTCAACACCCTGTC |
| Rat-Myocardin | CCAACAGTTCCGGAGATAACC | ACTTGGGGAGGATGGTGGTT |
| Rat-KLF4 | CCCCTCTCTCTTCTTCGGACTC | CCTGGTGGGATAGCGAGTTGGA |
| Rat-mmp9 | GATCCCCAGAGCGTTACTCG | GTTGTGGAAACTCACACGCC |
| Rat-mmp2 | AGAAGGCTGTGTTCTTCGCA | AAAGGCAGCGTCTACTTGCT |
| Rat-il1β | AGGCTTCCTTGTGCAAGTGT | TGAGTGACACTGCCTTCCTG |
| Rat-il6 | TGTGCAATGGCAATTCTGAT | GAGCATTGGAAGTTGGGGTA |
| Rat-il18 | ATATCGACCGAACAGCCAAC | ATCCCCATTTTCATCCTTCC |
| Rat-gapdh | GCACCGTCAAGGCTGAGAAC | TGGTGAAGACGCCAGTGGA |

**
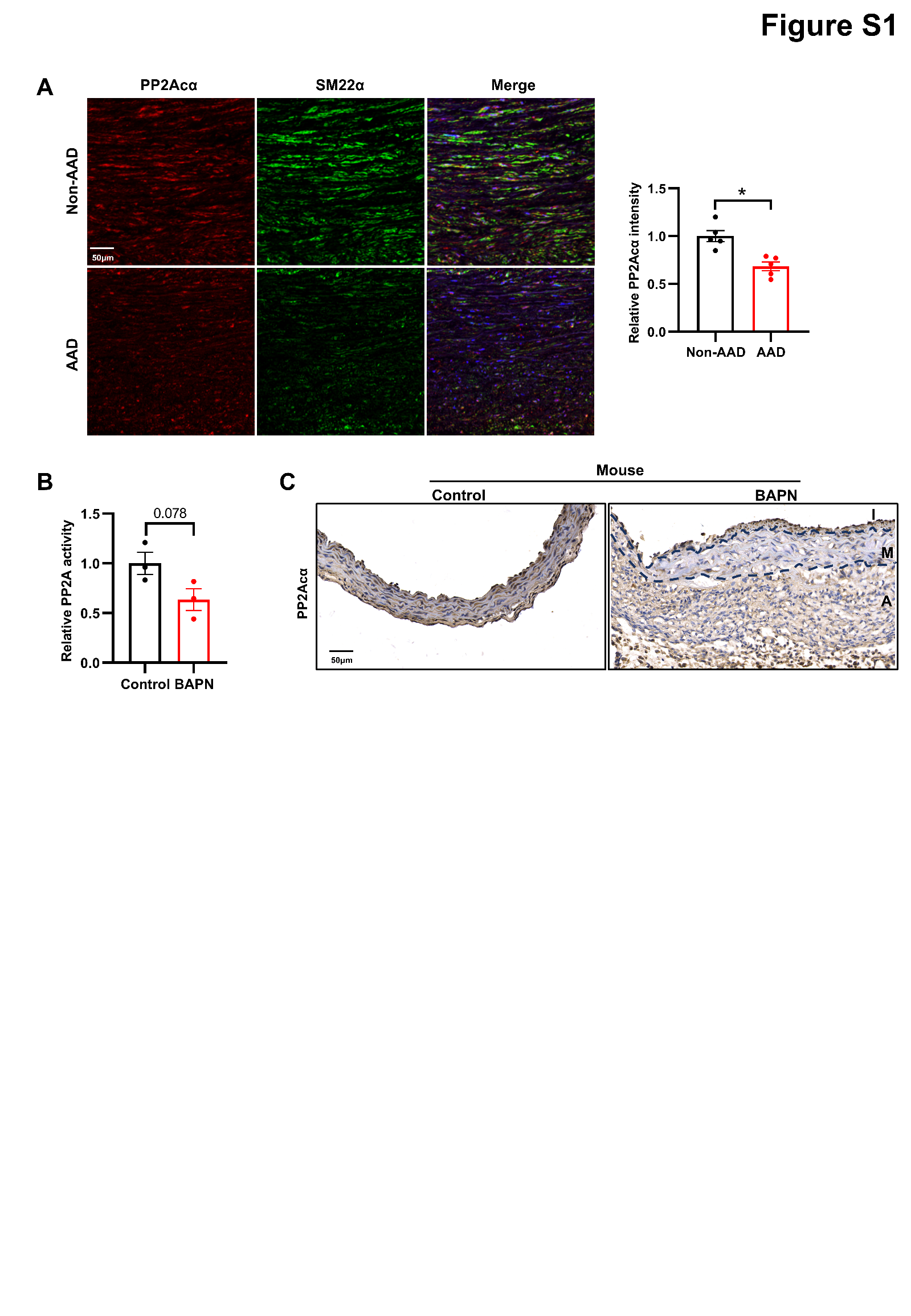
**

**Fig. S1 PP2A activity and PP2Acα expression are reduced in the aortas of BAPN-treated mice.** (A) Representative images of triple immunofluorescence for PP2Acα, SM22α, and DAPI, and quantification of PP2Acα expression in the human AAD and non-AAD segments. N=5, Student’s *t* test. (B) PP2A activity in mouse aortas after treating with BAPN (0.5% in drinking water) for 2 weeks. N=3, Student’s *t* test. (C) Representative images of PP2Acα expression by immunohistochemistry staining of mouse aortas after BAPN treatment for 4 weeks. **P*<0.05.


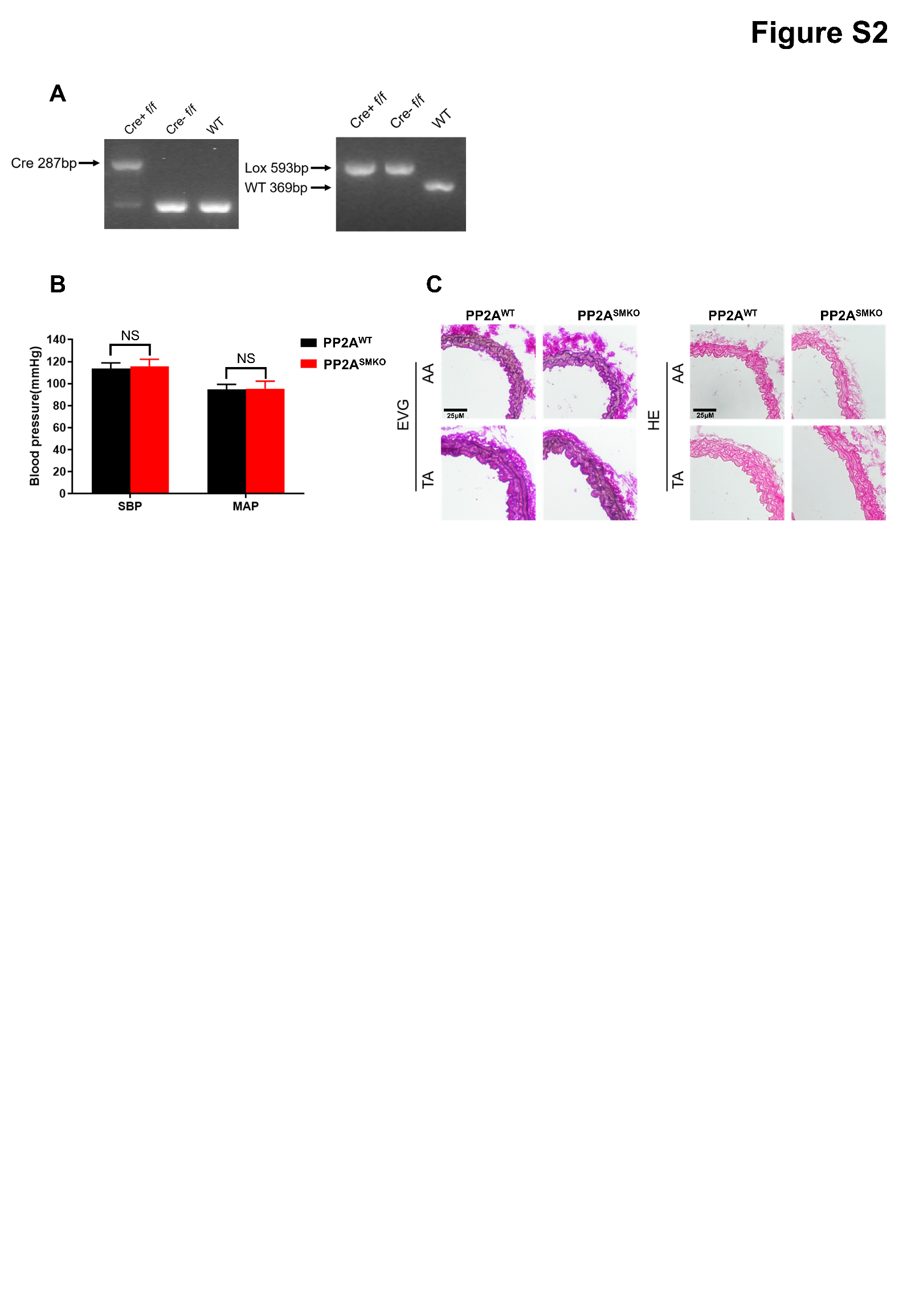
**Fig. S2. Genotyping, blood pressure results and arterial morphology of PP2A^SMKO^ mice.** (A) Genotyping results of Myh11 Cre^+/−^, PP2Acα^flox/flox^ mice; Myh11 Cre^−/−^, PP2Acα^flox/flox^ mice and WT mice. (B) Systolic blood pressure (SBP) and mean arterial pressure (MAP) in conscious 12-week-old PP2A^WT^ and PP2A^SMKO^ mice using the tail-cuff method. N=5, Student’s *t* test. (C) Representative images of histological staining with HE and EVG in thoracic and abdominal aortic sections of 12-week-old PP2A^WT^ and PP2A^SMKO^. Scale bars=25 μm. NS means no significant difference.


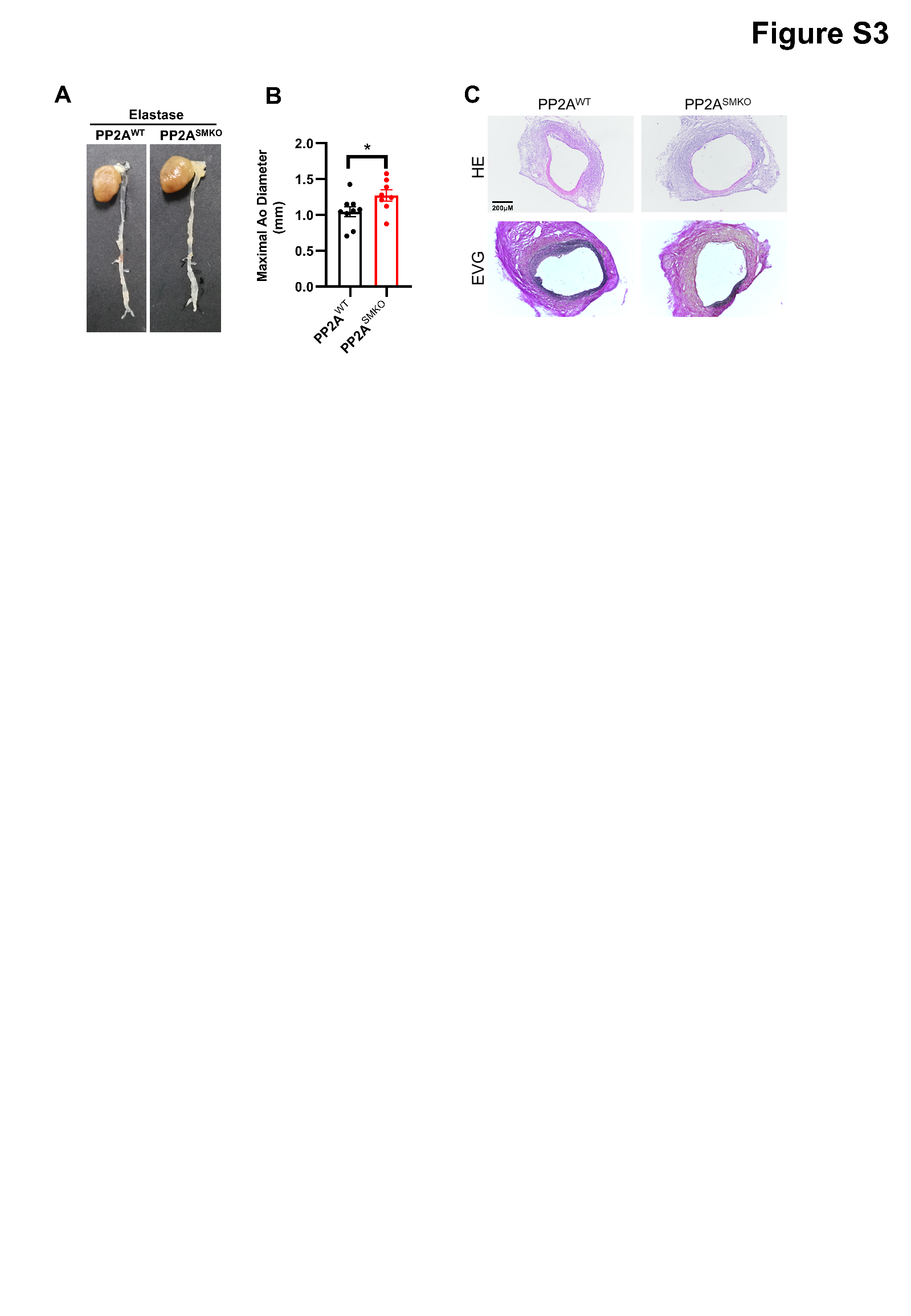
**Fig. S3. PP2Acα deficiency in VSMCs aggravates Elastase-induced AAA development.** (A) Representative pictures of whole aortas of PP2A^WT^ and PP2A^SMKO^ mice after elastase treatment for 14 days. (B) Maximal abdominal aortic diameters of PP2A^WT^ and PP2A^SMKO^ mice after elastase treatment for 14 days. N=8-9, Student’s *t* test. (C) Representative images of histological staining with HE and EVG in the abdominal aortic sections of 12-week-old PP2A^WT^ and PP2A^SMKO^ after elastase treatment for 14 days. **P*<0.05.


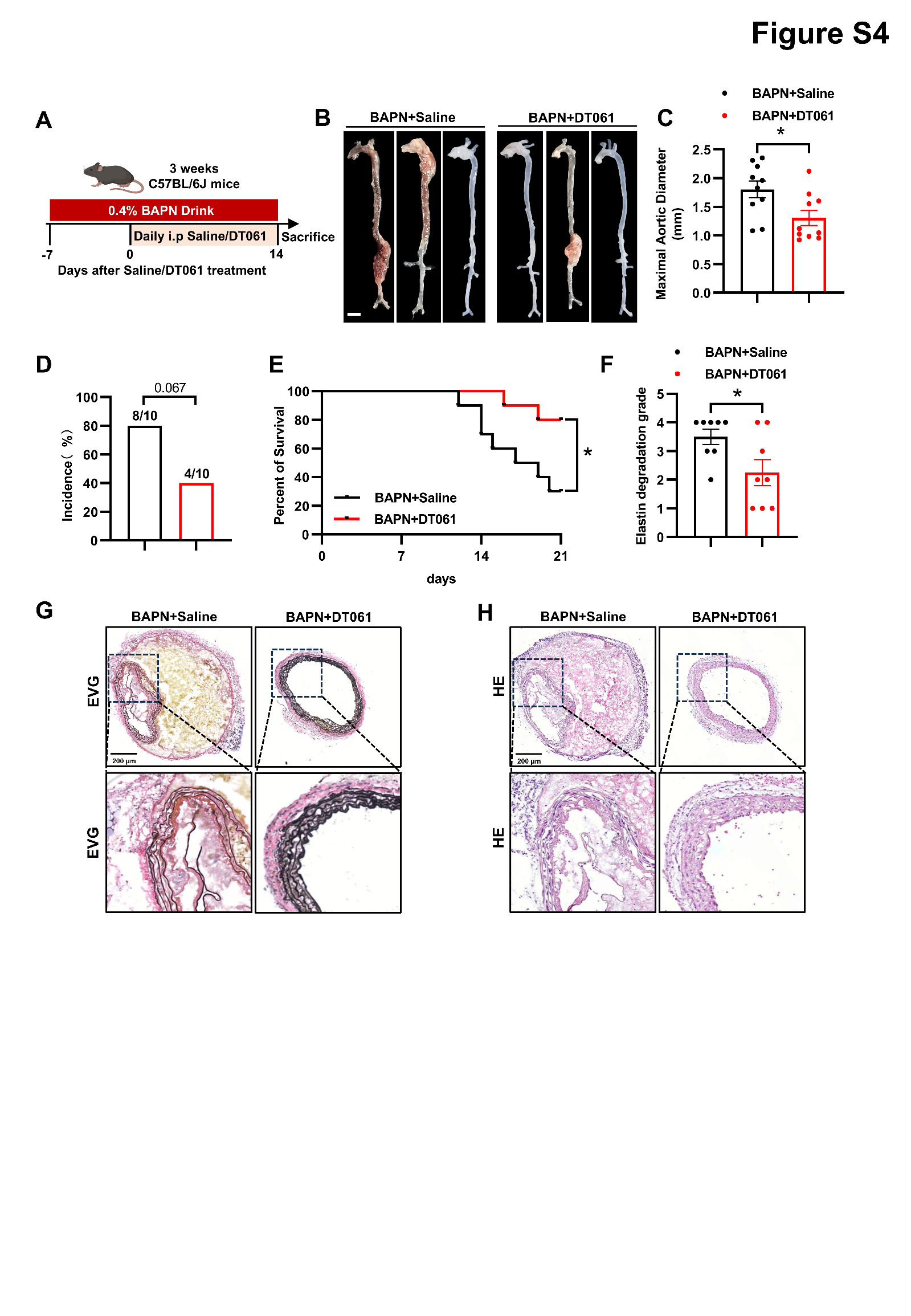


**Fig. S4. DT061 attenuates BAPN-induced AAD development.** (A) Experimental design. Three-week-old C57BL/6J mice were fed with BAPN (0.4% in drinking water) for 7 days, followed by daily intraperitoneal injections of DT061 (PP2A agonist, 3 mg/kg/day) and continued treatment for another 14 days until the end of the experiments. (B) Representative pictures of whole aortas of BAPN-fed mice with or without DT061 treatment. (C) Maximal aortic diameters, (D) Aneurysm incidence, (E) The survival curves of BAPN-fed mice with or without DT061 treatment. Scale bars=2 mm. N=10, Student’s *t* test for panel C, Chi-square test for panel D, Kaplan-Meier method and compared by log-rank tests for panel E. (F) Elastin fragmented score in the thoracic aortas from BAPN-fed mice with or without DT061 treatment. N=8, Mann-Whitney test. (G) Representative images of histological staining with hematoxylin and eosin (HE) and (H) Verhoeff’s Van Gieson (EVG) in aortic sections of BAPN-fed mice with or without DT061 treatment. Scale bars=200 μm. **P*<0.05.


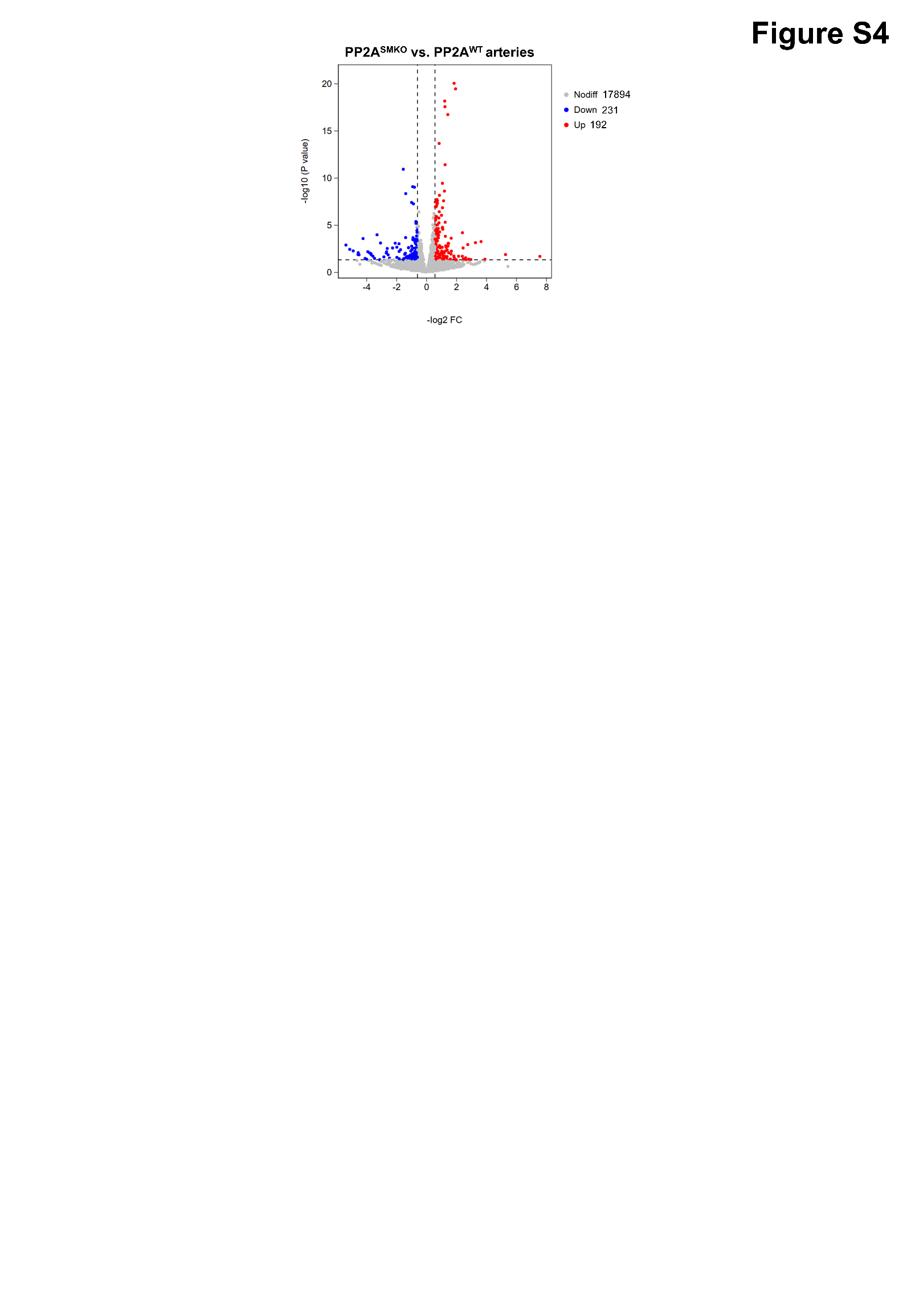
**Fig. S5. Differential gene expression between PP2A^SMKO^ and PP2A^WT^ mouse aortic tissues.** Volcano plot with up- or down-regulated genes in aortas from PP2A^SMKO^ mice highlighted in red or green, respectively.

**
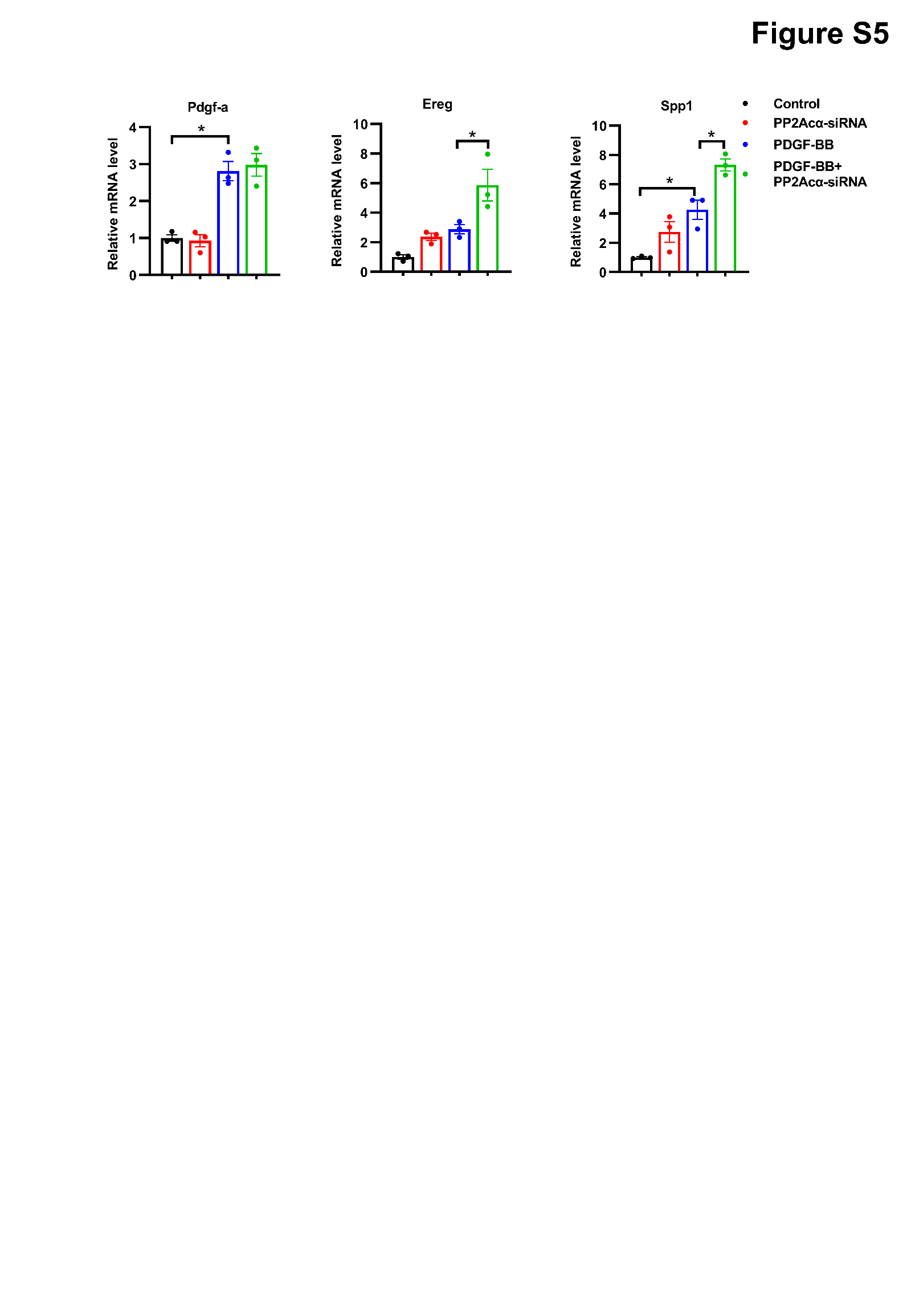
Fig. S6. PP2A deficiency enhances the PDGF-BB-stimulated upregulation of secretory VSMC markers.** VSMCs were transfected with the Scr-siRNA or PP2Acα-siRNA (50 nM) for 24 h, then were treated with PDGF-BB (20 ng/mL) for 12 h. The quantitative analysis of mRNA levels of Pdgf-a, Ereg and Spp1. N=3, two-way ANOVA followed by the Bonferroni test. **P*<0.05.


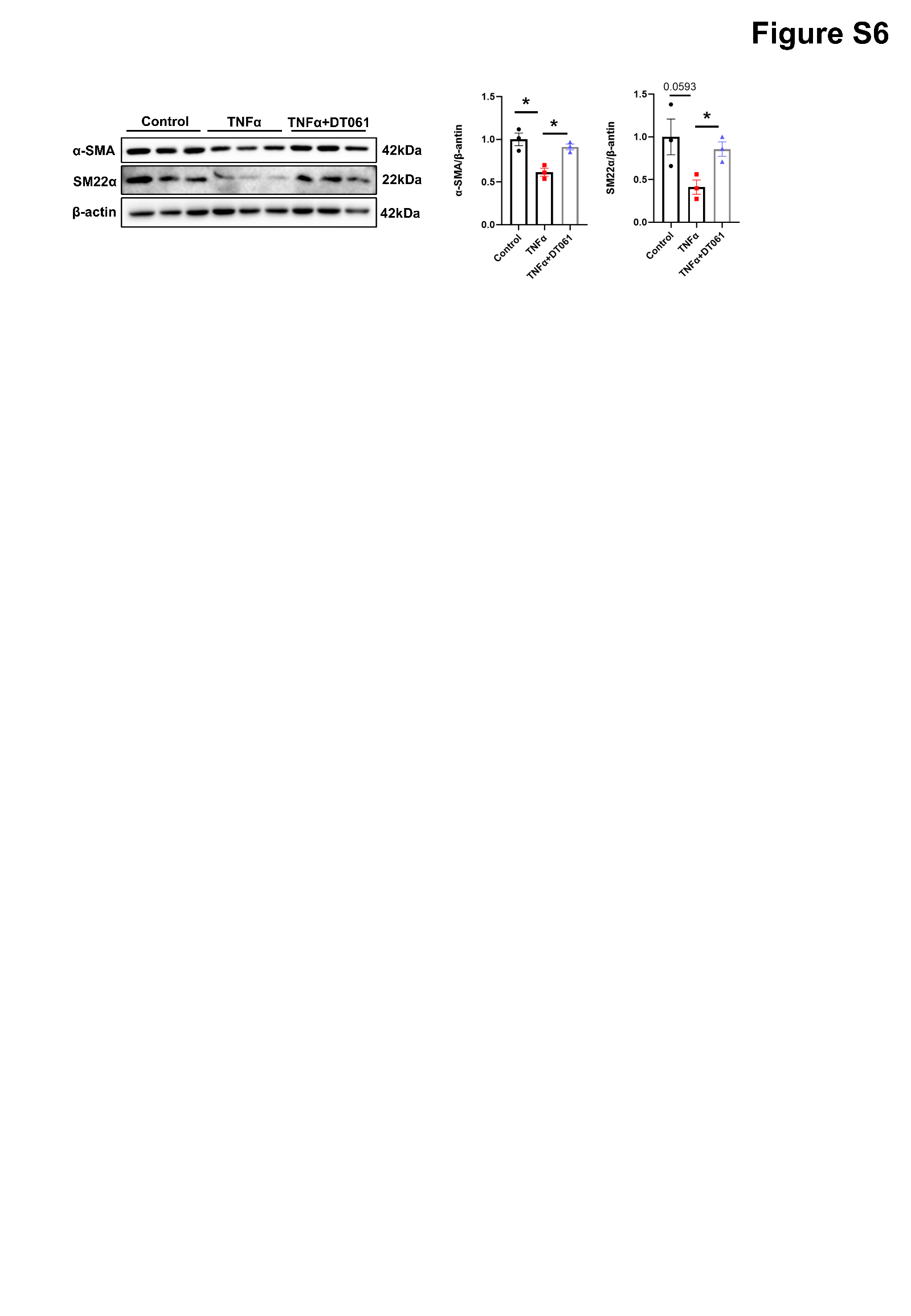
**Fig. S7. PP2A agonist inhibits TNFα-induced VMSC dedifferentiation.** VSMCs were pre-treated with PP2A agonist DT-061 (10 μM) for 1 h, and then treated with TNFα (20 μg/mL) for 24 h. Representative Western blot images and quantification analysis of α-SMA and SM22α. N=3, one-way ANOVA followed by the Tukey’s test. **P*<0.05.


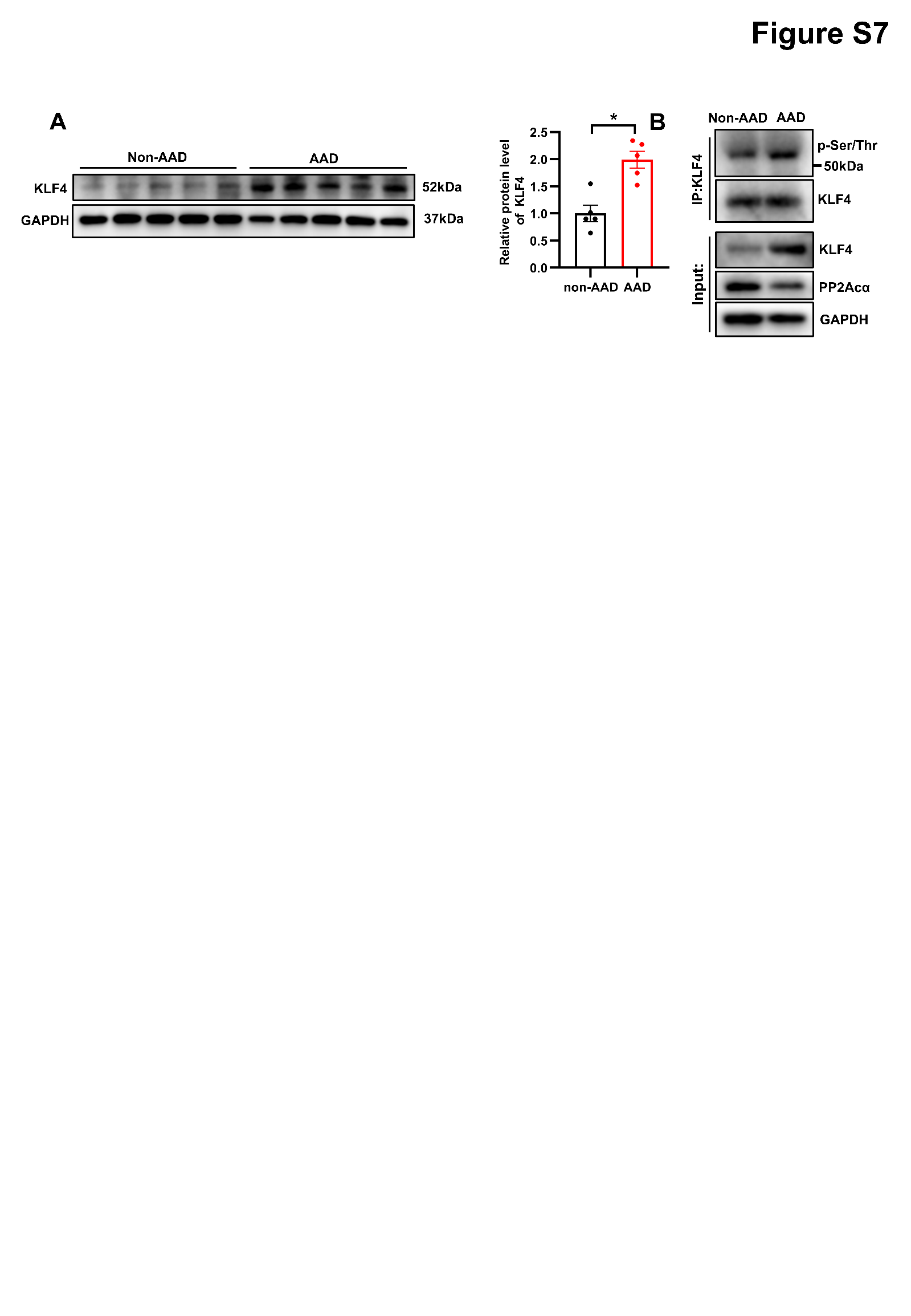
**Fig. S8. KLF4 protein and Ser/Thr phosphorylation level increased in human AAD tissues.** (A) Representative Western blot images and quantification of KLF4 in aortic tissues from non-AAD and AAD patients. N=5, Student’s *t* test. (B) Immunoblotting using phospho-Thr/Ser-specific antibody to detect phosphorylated KLF4 level after immunoprecipitation with KLF4 in the aortic tissues from non-AAD and AAD patients. A representative blot is shown, N=3. **P*<0.05.

**
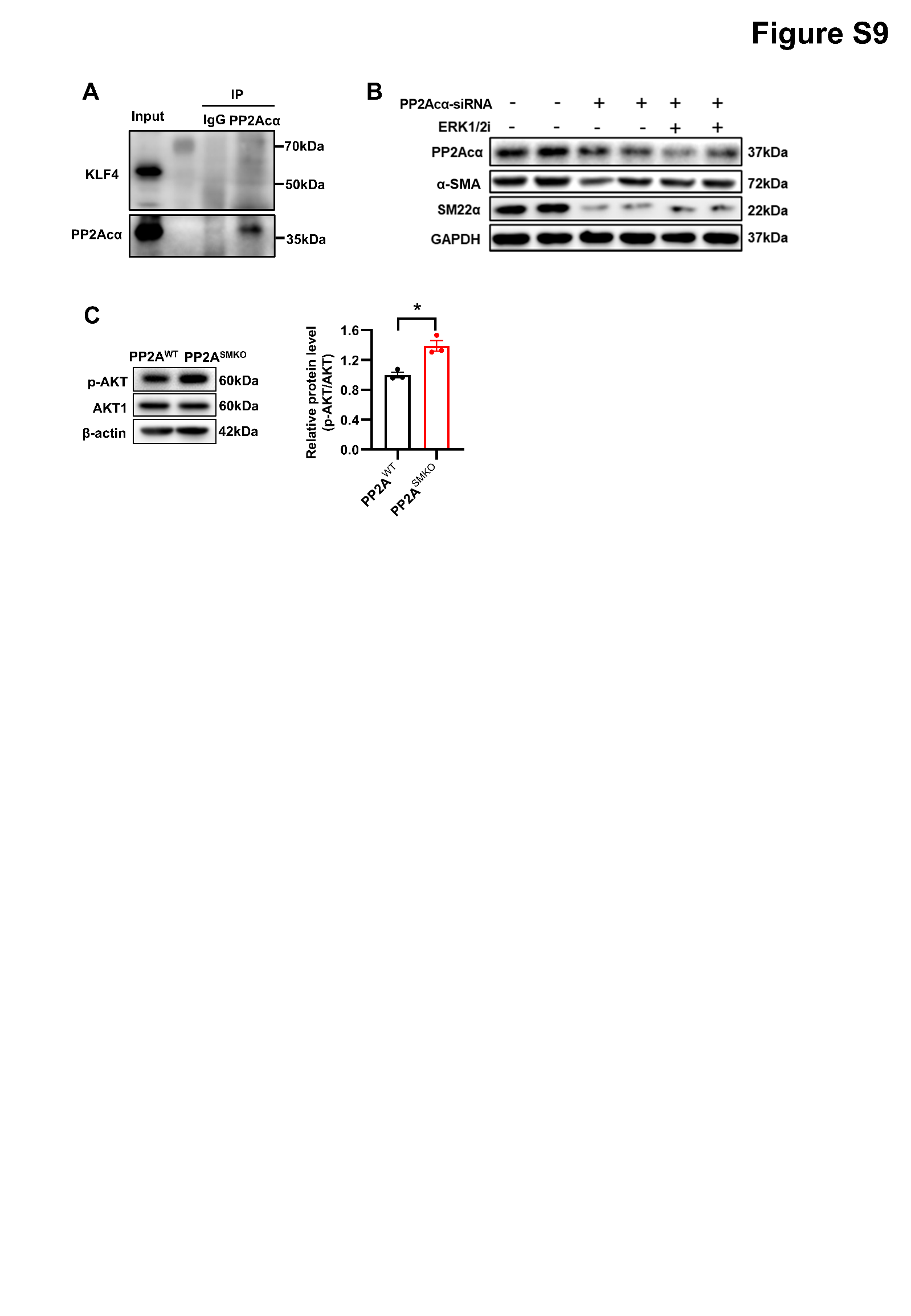
Fig. S9. ERK inhibitor does not prevent the decreased level of contractile marker proteins induced by PP2Acα deficiency.** (A) Endogenous coimmunoprecipitation assay in VSMCs. Cell extracts were immunoprecipitated with an anti-PP2Acα antibody or control IgG (immunoglobulin G) and analyzed by Western blot with an anti-KLF4 antibody. A representative blot is shown, N=3. (B) Representative Western blot images of PP2Acα，α-SMA and SM22α in VSMCs transfected with PP2Acα-siRNA (50 nM) for 6 h and then treated with ERK1/2i (ERK1/2 inhibitor, U0126, 1 μM) for 48 h. N=3. (C) Representative Western blot images and quantification of p-AKT in aortas from PP2A^WT^ and PP2A^SMKO^ mice. N=3, Student’s *t* test.


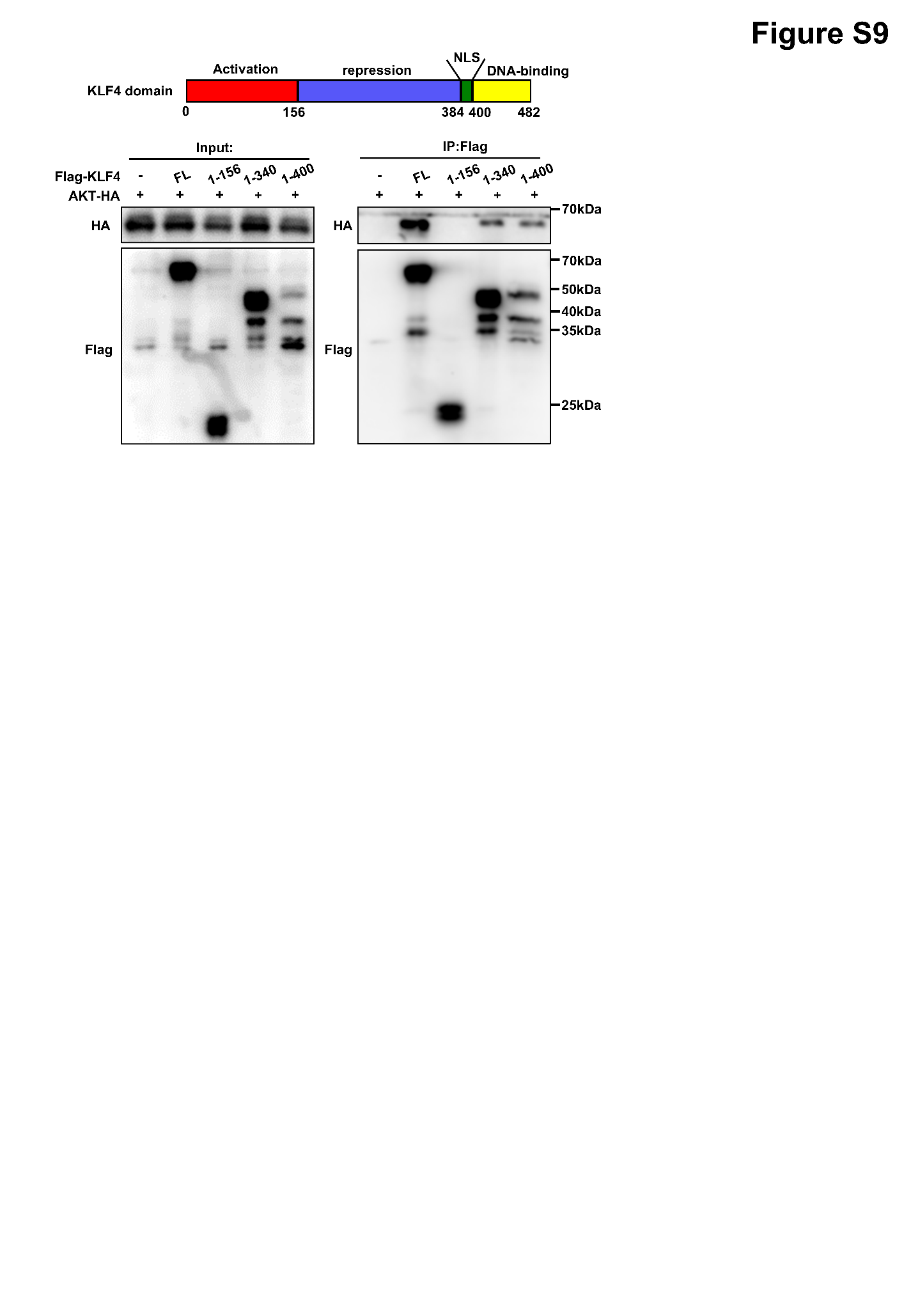
**Fig. S10. C-terminal domain of KLF4 interacts with AKT1.** HEK293T cells were co-transfected with the HA-AKT1 plasmid and serial deletion mutants of FLAG-KLF4. Proteins from cells were immunoprecipitated with the FLAG antibody, and the precipitates were analyzed using the HA antibody. A representative blot is shown, N=3.

**
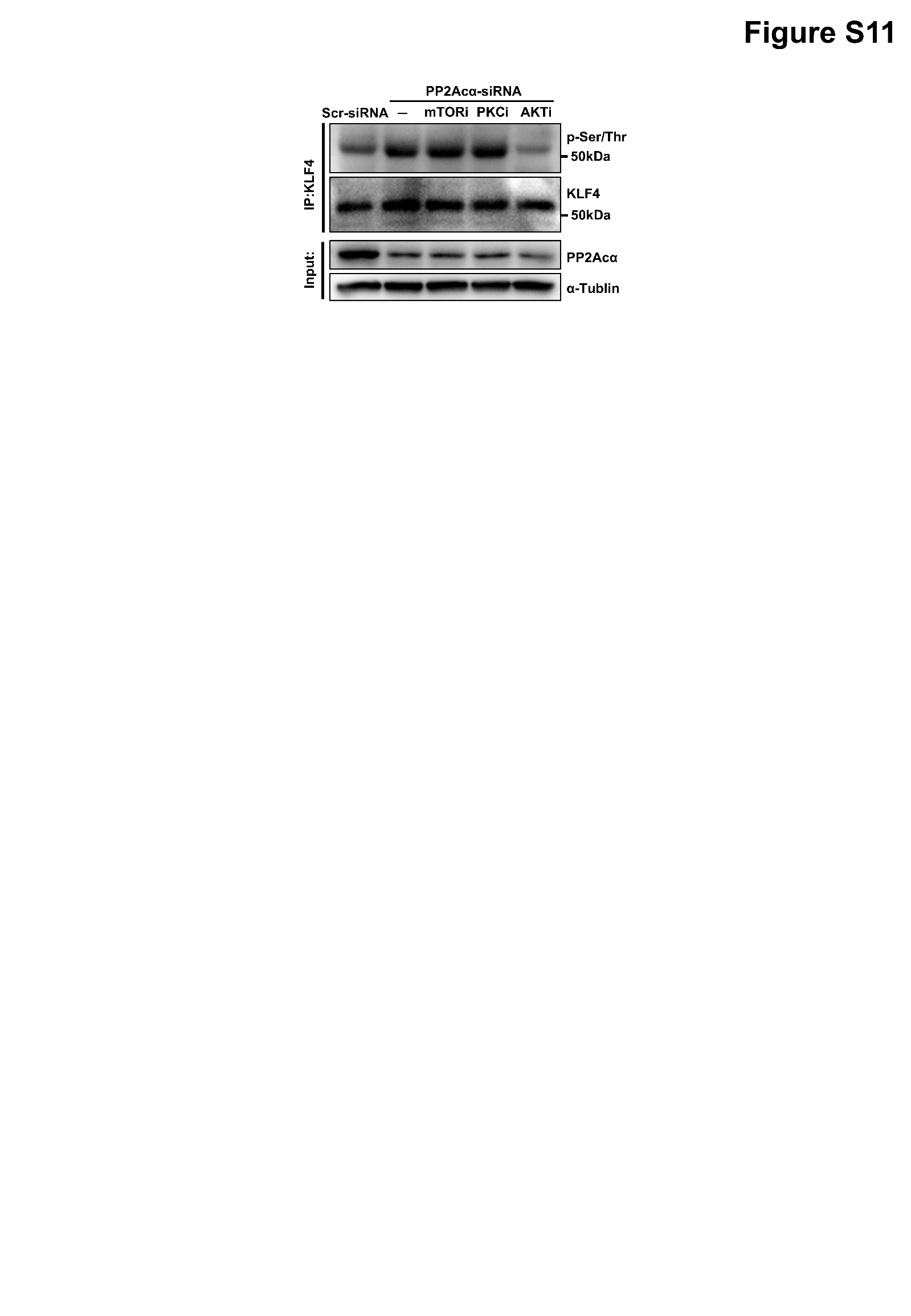
Fig. S11. mTOR and PKC inhibitors do not affect the upregulated phosphorylation of KLF4 induced by PP2A knockdown.** VSMCs were transfected with PP2Acα-siRNA (50 nM) for 6 h and then treated with mTORi (mTOR inhibitor, rapamycin, 100 nM), PKCi (PKC inhibitor, Go6983, 1 μM) or AKTi (AKT inhibitor, MK2206, 5 μM) for 48 h, and immunoblotting using phospho-Thr/Ser-specific antibody to detect phosphorylated KLF4 level after immunoprecipitation of KLF4 from VSMCs. A representative blot is shown, N=3.

.
